# Microglial Cofilin-1 Regulates Neuroinflammatory Responses and Behavioral Outcomes Following Intracerebral Hemorrhage

**DOI:** 10.1007/s12035-026-06324-7

**Published:** 2026-11-09

**Authors:** Daniyah A. Almarghalani, Mohammad Ali, Marco B. Rust, F. Scott Hall, Zahoor A. Shah

**Affiliations:** 1 Department of Pharmacology and Experimental Therapeutics, College of Pharmacy and Pharmaceutical Sciences, The University of Toledo, Toledo, OH USA; 2 Department of Pharmacology and Toxicology, College of Pharmacy, Taif University, PO Box 11099, 21944 Taif, Saudi Arabia; 3 Department of Medicinal and Biological Chemistry, College of Pharmacy and Pharmaceutical Sciences, The University of Toledo, Toledo, OH USA; 4 Molecular Neurobiology Group, Institute of Physiological Chemistry, Center for Mind, Brain and Behavior (CMBB), University of Marburg, University of Marburg and Justus-Liebig-University Giessen, 35032 Marburg, Germany

**Keywords:** Cofilin-1, Microglia, Sex differences, Intracerebral hemorrhage, Behavioral phenotypes, Neuroinflammation

## Abstract

Cofilin-1, an actin-binding protein, modulates cytoskeletal dynamics and cellular motility. Although its role in conditional neuron knockout (KO) and neuronal injury has been well studied, the specific deletion of cofilin-1 in microglia and its role in neuroinflammation after intracerebral hemorrhage (ICH) remains unknown. This study aimed to characterize the effects of microglia-specific cofilin-1 deficiency on microglial morphology, homeostasis, behavioral phenotypes, and neurobehavioral and neuroinflammatory outcomes following ICH in male and female mice. A conditional KO mouse model was generated by crossing cofilin^fl/fl^ (Flx) mice with *CX3CR1-Cre* drivers to achieve microglia-specific deletion. Baseline behavioral performance, microglial morphology, and homeostatic markers were evaluated in both sexes. ICH was then induced by intra-striatal collagenase injection, and neurobehavioral function, brain edema, hematoma volume, microglial activation, and peripheral immune responses were evaluated three days post-injury. Microglial deletion of cofilin-1 was associated with reduced TMEM119 expression, increased IBA1 immunoreactivity, and morphological transformation toward an activated phenotype. Female KO mice showed pronounced hyperlocomotion and decreased anxiety-like behavior, whereas both sexes exhibited impaired neuromuscular performance in the wire hang test. After ICH, KO mice had significantly larger hematoma volumes, enhanced microglial activation, and increased peripheral leukocyte counts compared with Flx controls. Notably, these changes were more severe in female KO mice, indicating a sex-dependent vulnerability. Our findings establish cofilin-1 as a key regulator of microglial homeostasis and immune balance within the brain. Deficiency of microglial cofilin-1 predisposes to worse secondary brain injury after ICH in a sex-dependent manner.

**Supplementary Information:**

The online version contains supplementary material available at https://doi.org/10.1007/s12035-026-06324-7.

## Introduction

Intracerebral hemorrhage (ICH) accounts for 10–15% of strokes worldwide; however, it is associated with high mortality and disability. ICH pathophysiology is divided into two phases: primary and secondary injury. Primary injury is related to hematoma-induced mass effects and physical disruption of brain tissue. In contrast, secondary injury is accompanied by pathological responses due to hematoma, which include oxidative stress, iron toxicity resulting from hemoglobin breakdown, disruption of the blood–brain barrier (BBB), cell death, neuroinflammation, and microglial activation [1, 2]. Microglia, the brain’s resident macrophages, are among the earliest responders to ICH, where they rapidly transition from surveillant phenotypes (M0) to activated phenotypes that can either exacerbate tissue damage (M1 phenotypes; morphological and inflammatory changes consistent with a more pro-inflammatory activation state) by releasing chemokines, cytokines (e.g., tumor necrosis factor-α (TNFα), interleukin-1β (IL-1β)- and IL-6) and reactive oxygen species (ROS), or promote repair (M2 Phenotypes) through phagocytosis of blood products and debris, release neurotrophic factors and tissue repair markers (e.g. IL-4, IL-10 and arginase − 1), and stimulate angiogenesis [2–5]. Actin cytoskeleton dynamics are fundamental to microglial motility, process remodeling, phagocytosis, and immune signaling. Among the core regulators of actin turnover is cofilin-1, an actin-binding protein mainly expressed in the central nervous system that severs and depolymerizes F-actin to accelerate filament turnover and drive cell motility and shape changes [6, 7]. Dysregulation of cofilin-1 signaling has been implicated across neurological injury and neurodegenerative diseases (e.g., Alzheimer’s disease, Huntington’s disease, Parkinson’s disease, schizophrenia, ischemic stroke,ICH, and traumatic brain injury), through mechanisms such as cofilin–actin rod formation, mitochondrial translocation, oxidative stress, inflammatory response and apoptosis signaling [7–10].

In microglia, perturbation of cofilin-1 activity may alter main effector functions. For example, knockdown of cofilin-1 in microglial cell lines using siRNA reduced LPS-induced activation, proliferation, migration, and chemotaxis, as well as decreased the production of inflammatory mediators through the NF-κB and JAK-STAT pathways [11]. Studies further showed that acute treatment with either a cofilin-1 siRNA [12] or a cofilin inhibitor [13] after ICH improved behavioral and memory performance, promoted hematoma resolution, increased presynaptic and postsynaptic markers, while reducing cofilin overexpression, cofilin-actin rod formation, microglial activation, neuroinflammation, and neuronal death [12, 13]. Thus, cofilin-1 plays an essential role in secondary brain injury following ICH and may serve as a potential therapeutic target, particularly when inhibited in the acute phase after ICH. However, conditional knockout of cofilin-1 in neurons of the forebrain using CaMKII-Cre results in abnormal dendritic spine morphology, accumulation of synaptic F-actin, and reduced AMPA receptor diffusion in extrasynaptic domains and cognitive impairments [14], leading to deficits in associative learning, including impaired spatial learning, fear conditioning, and reward-based learning [14], as well as a decrease in anxiety-related behavior [15]. Double mutants, knockout of ADF and cofilin-1, reveal more severe presynaptic defects affecting vesicle organization and exocytosis [16], along with ADHD-like behavioral abnormalities, including increased locomotor activity and working memory deficits [17]. These findings suggest that while transient suppression of overexpression and activation of cofilin-1 post-ICH is neuroprotective, the conditional and complete loss of cofilin-1 impairs important neuronal structure and multiple behavioral functions.

Preclinical studies suggest that microglial phenotypes and neuroinflammatory responses after ICH are strongly influenced by sex. Female mice consistently demonstrate improved neurobehavioral recovery relative to males, despite similar acute lesion progression following ICH [18]. Xie et al. demonstrated that hematoma volume and cerebral edema in the first 24 h did not differ significantly by sex [19]. However, female mice showed substantially better neurobehavioral recovery over a 30-day assessment period [19]. In addition, transcriptomic and proteomic patterns indicated a sex-specific acute-phase response, with female-specific pathways enriched in cell development, growth, and proliferation. In contrast, male-specific pathways were primarily involved in molecular degradation [19]. Hormonal modulation further influences outcomes; estrogen and progesterone provide neuroprotection, while testosterone may exacerbate injury [20, 21]. A clinical study reported that men have a higher incidence of ICH and at younger ages compared to women, with differences in bleed location and comorbidity that may relate to differences in immune responses [22]. We therefore suggested that microglial cofilin-1 deficiency would reprogram microglial morphology, motility, and effector functions after ICH, and that these effects would be sex-specific. We developed and characterized cofilin-1 deficiency mainly in microglia in male and female mice in the context of homeostasis and then in experimental ICH, offering a tractable framework to (i) define how cofilin-1 deficiency at baseline impacts the shape of microglia and influences the behavioral outcomes across sexes; (ii) determine the influence of cofilin-1 deficiency after ICH and assess behavioral performance, edema, hematoma volume, cofilin and microglial activation as well as blood inflammatory responses.

## Materials and Methods

### Mice

This study was conducted in accordance with the National Institutes of Health (NIH) guidelines for the use of experimental animals. Experimental protocols were approved by the University of Toledo Animal Care and Use Committee (approval number 108615). Adequate procedures and measures were taken to minimize the number of mice used and to ensure minimal pain and/or discomfort for the mice. Cofilin^fl/fl^ mice were obtained from Dr. Marco B. Rust, University of Marburg, Germany. CX3Cr1-Cre mice were acquired from Jackson Labs. Conditional deletion of the cofilin-1 gene in the microglia was achieved by crossing mice expressing floxed alleles (cofilin^fl/fl^, Flx) with a mouse strain that carries the Cre recombinase under the direction of the Cx3Cr1-Cre promoter in the microglia to produce the CX3Cr1^Cre^.cofilin^fl/fl^ (knockout, KO) mice (Supplementary Fig. 1 A). The genotype of mice was determined using PCR analysis of progeny ear punch DNA (Supplementary Fig. 1 B and Supplementary Table 1). To validate gene deletion, we isolated microglia from both Flx and KO mice using the magnetic bead technique (Miltenyi Biotec, RRID: SCR_008984). We then performed flow cytometry to confirm microglial purity. We also performed flow cytometry on isolated microglia with cofilin Alexa Fluor to confirm cofilin-1 deletion in microglia. In addition, we performed qPCR on isolated microglia from both Flx and KO mice. These approaches assessed reductions in cofilin-1 protein and mRNA expression, respectively. Although these findings support reduced cofilin-1 expression in the isolated cell fraction, they do not establish exclusive recombination in microglia or exclude recombination in other CX3CR1-expressing cell populations. For immunohistochemistry, we sacrificed Flx and KO mice to visualize cofilin expression and microglial morphology and counts.

### Tissue preparation

The mice underwent a perfusion procedure to isolate the brain for flow cytometry and immunohistochemistry. In flow cytometry, anesthesia (Ketamine/xylazine) was administered intraperitoneally at doses of 90–120 mg/kg and 10 mg/kg, followed by transcardial perfusion with 1X phosphate-buffered saline (PBS), and then the brains were collected. For immunohistochemistry, anesthesia was performed using (Ketamine/xylazine) for transcardial perfusion with cold 1X PBS, followed by perfusion with cold 4% paraformaldehyde (PFA) in PBS for fixation. Brains were removed and post-fixed overnight in 4% PFA. Coronal brain Sects. (5 μm thickness) were prepared using a microtome and collected for immunofluorescence staining.

### Behavioral Characterization

**Fig. 1:**
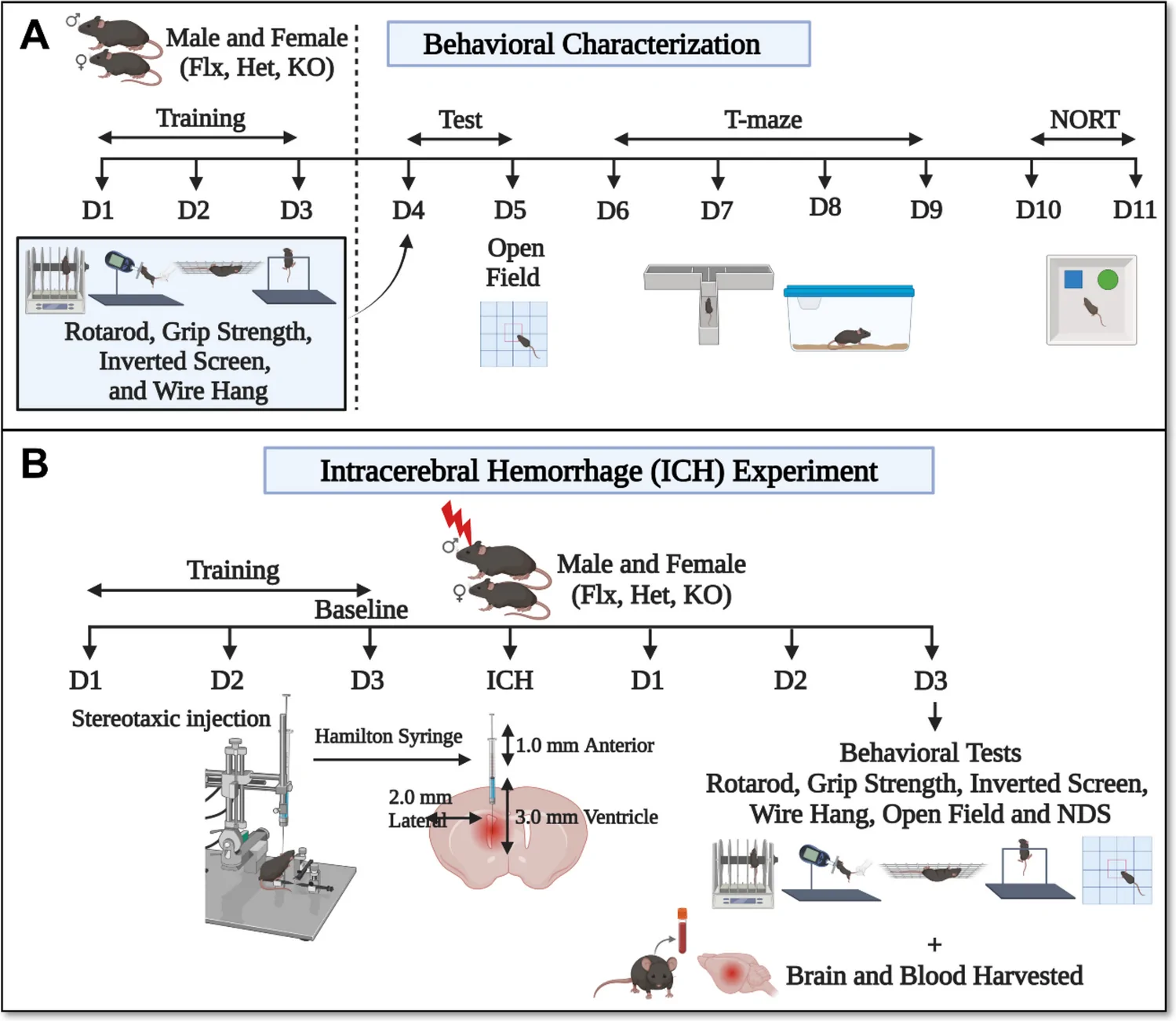
Characterization and experimental design for behavioral assessment and ICH) induction in cofilin-1 KO mice. (**A**) Behavioral characterization: Schematic representation of the experimental timeline for behavioral testing in male and female cofilin^fl/fl^ (Flx), CX3Cr1^Cre^.cofilin^fl/wt^ (Het) and CX3Cr1^Cre^.cofilin^fl/fl^ KO mice. Animals underwent a 3-day training phase (D1–D3) for rotarod, grip strength, inverted screen, and wire hang tasks. Testing began on day 4 (D4) with the rotarod, grip strength, inverted screen, and wire hang performance assessment, followed by an open-field evaluation on day 5 (D5). Spatial working memory was assessed using the T-maze on days 6–9 (D6–D9), and recognition memory was tested using the novel object recognition test (NORT) on days 10–11 (D10–D11). A total of 84 mice were used. For behavioral tests, male: Flx (n = 10), Het (n = 10), KO (n = 12); female: Flx (n = 10), Het (n = 10), KO (n = 14); flow cytometry, Flx (n = 3) and KO (n = 3); immunohistochemistry, Flx (n = 6) and KO (n = 6). (**B**). ICH experimental design: A total of 66 mice were divided into three groups, Flx + ICH, Het + ICH, and KO + ICH (n = 10- 14 in each group of males and females). First, mice were tested on the rotarod, grip strength, inverted screen, and wire hang tests for 3 days and 24 h before the ICH, which was considered the baseline (each mouse was tested 3 times per trial). Different cohorts of mice were subjected to intra-striatal collagenase injection-induced ICH. After the ICH, we performed neurobehavioral tests (rotarod, grip strength, wire hang, inverted screen, neurological deficit score (NDS), and open field test. Behavior was scored for all groups and compared among groups 24 h before ICH and on day 3 after ICH. On day 3, animals were euthanized, and the brain and blood were collected for further biological assessment

A total of 84 male and female mice of three genotypes, including cofilin^fl/fl^ (control/floxed, Flx), CX3Cr1^Cre^.cofilin^fl/wt^ (heterozygous, Het), and CX3Cr1^Cre^.cofilin^fl/fl^ (knockout, KO), aged 6–8 months, were used for behavioral assessments. Mice aged 6- 8 months were selected to represent fully mature adult animals while avoiding age-related neurodegenerative changes observed in older mice. This age range also ensured the availability of sufficient age-matched littermates generated through the breeding strategy required for the conditional knockout colony. All experiments were conducted during the light phase under standard housing conditions (12-h light/dark cycle, with ad libitum access to food and water). Behavioral testing was performed over 11 consecutive days. Mice first underwent a 3-day training phase (D1–D3) on the rotarod, grip strength, inverted screen, and wire hang tasks to familiarize them with the procedures. We maintained a minimum 4-h gap between behavioral experiments to ensure adequate recovery time. On day 4 (D4), motor coordination and strength were evaluated using rotarod, grip strength, inverted screen, and wire hang tests. On day 5 (D5), we assessed locomotor activity and anxiety-like behavior in the open-field test. From day 6 to day 9 (D6–D9), spatial working memory was evaluated using the T-maze spontaneous alternation test. On days 10 and 11 (D10–D11), recognition memory was assessed using the novel object recognition test (NORT). All behavioral scoring was performed by investigators blinded to genotype and sex (Fig. 1A shows the flowchart of the experimental design).

### ICH Model and Experimental Design

Male and female Flx, Het, and KO mice (n = 66; aged 6 to 8 months) were used. All animals were trained for behavioral tasks before ICH (rotarod, grip strength, inverted screen and wire hang) and baseline performance was recorded. Next, all groups were subjected to intra-striatal collagenase injection-induced ICH using a stereotaxic frame (Stoelting Lab Standard Stereotaxic Instrument with Mouse Adaptor, Wood Dale, IL). Isoflurane was used to induce anesthesia, and a small hole was made in the skull above the left striatum of the mice. A Hamilton syringe needle was then inserted to deliver collagenase (0.6 µl, 0.09 units). The stereotaxic coordinates used for collagenase injections are located at a position 1 mm anterior to the bregma, 2 mm lateral to the brain midline, and 3 mm deep below the skull surface. The needle was slowly withdrawn (after 10 min of slow injection to stop reflow), and the wound was sutured. The mice were placed in an incubator for post-surgical recovery. Mice were transferred to clean cages with free access to water and food and survived for 3 days. Following the injury, behavioral assessments were conducted on day 3, including the rotarod, grip strength, inverted screen, wire hang, open-field test, and neurological deficit scores (NDS). A minimum of four hours was maintained between behavioral experiments to allow the animals sufficient time to recover. After that, the animals were euthanized, followed by collecting the brain and blood samples for histological and biochemical analyses. Mice underwent decapitation to dissect brains for brain edema evaluation. For hematoma volume and immunohistochemistry, mice were euthanized, and perfusion was performed using cold 1X PBS and cold 4% PFA, as described in the perfusion technique above. The brains were then isolated. (Fig. 1B shows the experimental design of the ICH study).

### Neurobehavioral Tests

All neurobehavioral tests were completed by an experienced investigator who was blinded to the experimental groups.

#### Rotarod

The rotarod is used to evaluate equilibrium behavior and deficits in motor coordination after ICH, as previously described [12, 13, 23]. The mouse was placed on a moving rod of the rotarod, which was initially set to rotate at 1 round per minute (RPM). The speed was accelerated by 1 RPM every 10 s until the mouse fell off. The trial ended once the mouse fell from the rod, and the time spent on the rod was recorded. The animals underwent rotarod training 3 days before the ICH, which was considered the baseline (each animal was assessed 3 times per trial, and we used the average value).

#### Grip Strength Test

This test is used to measure the strength of the mouse’s forelimb muscles after ICH [12, 13]. The test was performed by placing the animal’s forelimb on a pull bar assembly and holding it by the tail. The maximum strength applied by the animal was shown on a digital screen and recorded (each mouse was tested 3 times per trial and the average value of the three trials was determined).

#### Kondziela’s Inverted Screen Test

The test is used to evaluate muscle strength using all four limbs [24]. The mouse was placed in the middle of the wire mesh screen, and then the screen was rotated to an inverted position. The screen was then held steadily 50 cm above a padded surface. The time (seconds) was recorded when the mouse fell off or when 60 s was reached (each mouse was evaluated 3 times per trial, and the average value of the three trials was calculated).

#### Wire Hang Test

The test is used to assess neuromuscular performance; specifically, motor latency was recorded when the mouse fell off. The duration of the mouse’s grip on the wire was recorded in seconds. The test was terminated when the 60-s time limit was reached. The test was repeated three times, and the average time of the three trials was determined.

#### Neurological Deficit Scoring (NDS)

NDS is a 28-point system used to evaluate sensory and motor deficits following ICH [25]. Seven tests (body symmetry, gait, climbing, circling behavior, whisker response, compulsory circling, and front limb symmetry) are graded from 0 (no deficit) to 4 (severe deficiency) and are assessed on day 3 following ICH.

#### Open Field Test

General locomotion, exploratory activity, and anxiety-like behaviors were determined using an open-field test [26]. Four square white acrylic open-top boxes (50 cm × 50 cm) were illuminated from above by a 60 W bulb. Four mice were analyzed at a time and placed individually in each box. The mice were initially kept in an unlit/dark testing room for 10 min before being placed in the center of each box for 30 min. The boxes were sanitized with 70% ethanol in between testing sessions. Behavior was examined from recordings made using an overhead camera and analyzed with Stoelting Any-Maze software (Wood Dale, IL). The total distance the mouse traveled, mobile time, average speed, and distance in the center were measured and analyzed.

#### Spontaneous Alternation

Spontaneous alternation is assessed in a T-Maze, an enclosed apparatus in the form of a “T” (central arm, 50 cm long; choice arms, 30 cm; all arms, 10 cm wide), used to evaluate the cognitive and learning abilities of mice [10, 13, 27]. During each trial, a mouse was initially placed in the start arm and given 2 min to explore both arms. Once the mouse selected a specific goal arm, that arm was physically blocked, and the mouse was left undisturbed for 30 s. Afterward, the mouse was returned to its cage for a 30-s interval, and the block in the maze was removed. The mouse was then placed back in the start arm with all arms accessible and allowed to choose an arm again. A correct choice was recorded when the second run differed from the first run within a given set, indicating alternation. Conversely, an incorrect choice was recorded when the mouse chose the same arm as in the previous run. This process was repeated twice a day over four days. The percentage of alternation was calculated based on these recorded choices.

% Alternation (Correct choice/Total set) X 100.

#### Novel Object Recognition Test

The novel object recognition test (NORT) is used to assess recognition memory and learning in mouse models of neurological disorders [28]. This test evaluates the spontaneous tendency of mice to spend more time exploring a novel object than a familiar one. The selection to explore the novel object reveals the use of recognition memory and learning. To conduct NORT, we followed a previous protocol with some modifications [25]. In brief, the mouse was placed in a box (50 cm × 50 cm) and habituated to exploring for 5 min. The next day, two identical novel objects (cube, 5 × 3 × 3 cm) were placed in the field, and the mouse was allowed to explore them for 5 min. After 3 h, we replaced the original object with a novel one (a yellow ball, 4 cm) and kept the familiar object (a cube). The mouse was then allowed unrestricted exploration for 10 min. A camera recorded the behaviors exhibited by each mouse in the trial and was evaluated using Stoelting Any-Maze software (Wood Dale, IL). A discrimination index and % total investigated time were calculated for each animal [29].

$$\begin{array}{c}Discrimination\;Index\;=\;(time\;with\;novel\;object\;-\;time\;with\;familiar\;object)/\;(time\;with\;novel\;object\;+\;time\;with\;familiar\;object)\\\%\;Total\;Investigated\;Time\;=\;(time\;with\;novel\;object)/\;(time\;with\;novel\;object\;+\;time\;with\;familiar\;object)\;\times\;100\end{array}$$

### Microglia Isolation

Brain tissues from Flx and KO mice were dissociated into single-cell suspensions with high cell viability using Miltenyi’s Neural Dissociation Kit (cat. No. 130–094–802), followed by the removal of myelin debris. Microglia were isolated from myelin-depleted cells of both groups using CD11b MicroBeads (Miltenyi Biotec, a specific marker for microglia, cat. No. 130–097–142) and immediately subjected to flow cytometry analysis to determine the number of positive CD11b^+^ (microglia) and negative CD11b^−^ (other cells) fractions.

### Flow Cytometry

CD11b^+^ and CD11b^−^ cells obtained from the isolation procedure were resuspended in buffer and immune-stained with anti-CD11b-PE (Miltenyi Biotec, cat. No. 130–110–553) or cofilin-Alexa Fluor (Abcam) antibodies for 10 min at 4 °C. After washing, the cells were centrifuged at 300 × g for 10 min. The supernatant was then removed, and the cell pellets were resuspended in the buffer for flow cytometer analysis. Flow cytometry was used to identify the fluorescence of stained cells at the following excitation/emission maxima: CD11b® Red: 566/574 nm and Alexa Fluor® 488 cofilin: 499/520 nm, using a flow cytometer BD FACSCanto™ (BD Biosciences, Becton–Dickinson, San Jose, CA). Data was analyzed utilizing FCS Express 7 plus De Novo software (Glendale, CA).

### qPCR

Quantitative qPCR was used to examine gene expression. TRIzol reagent (Invitrogen, Carlsbad, CA) was used to extract the total RNA from the isolated positive fraction (microglia) of cofilin-1 and KO mice. The iScript cDNA Synthesis Kit (Bio-Rad, catalog# 1,708,891, Hercules, CA) was used to synthesize the complementary DNA (cDNA), and SsoAdvanced Universal SYBR Green Supermix (Bio-Rad, catalog# 1,725,271) was used for qPCR. The 2^^ΔΔCT^ method was used to quantify gene abundance by comparing changes in the target gene to those in the control 18 S gene. Results were shown as fold changes relative to the control group. Primer sequences are provided in the supplementary data (**Supplementary Table 2)**.

### Brain Water Content or Edema Assessment

Mice were euthanized 3 days after ICH, and brains were removed and divided into three pieces (contralateral side, ipsilateral side, and cerebellum) to evaluate brain water content as previously described [30]. Brain tissues were weighed directly after sectioning to measure the wet weights. Subsequently, the samples were dried at 100 °C for 24 h to obtain dry weights. The percentage of water content was determined using the following formula:

$$Water\;content\;of\;brain\;tissue=\;\left[\left(wet\;weight\right)-\left(dry\;weight\right)/\left(wet\;weight\right)\right]\;X\;100\%$$

### Histology and Brain Hematoma Measurement

For these experiments, different groups of male and female mice (e.g., Flx, Het, and KO) were anesthetized on day 3 after ICH. Subsequently, the mice were transcardially perfused with cold 1X PBS, followed by cold 4% PFA, as described in the tissue preparation. The brains were then embedded in paraffin and sliced into Sects. (5 μm) using a rotary microtome. These brain sections were deparaffinized and hydrated using a graded series of methanol, and then subjected to Luxol fast blue/Cresyl violet staining to determine hematoma volume. Images were acquired using light microscopy connected to a Canon camera. The measurement of hematoma volume was performed using a previously published method [12, 13]. Additional sections were prepared for immunohistochemistry.

### Immunohistochemistry

Mouse brain sections were deparaffinized and subjected to an antigen retrieval process in a pressure cooker for 15 min. The sections underwent three consecutive 5-min washes with 1X PBS each, followed by blocking with 5% BSA dissolved in TBST for 2 h on a rocker. The primary antibodies, including mouse anti-cofilin (Abcam) at a dilution of 1:1000 and rabbit anti-IBA1 (Wako, Japan) at a dilution of 1:1000, were added and incubated overnight at a temperature of 4 °C. After 24 h, the slides were washed three times with 1X PBS, and the secondary antibodies were added. The slides were then incubated in a dark container for 1 h at room temperature, using Alexa Fluor donkey anti-mouse IgG (Jackson Immunoresearch, West Grove, PA, USA) at a dilution of 1:1000 for green labeling and Texas Red donkey anti-rabbit IgG (Jackson Immunoresearch) at a dilution of 1:1000 for red labeling. Following three washes with 1X PBS, the slides were mounted with DAPI (Molecular Probes, Eugene, OR, USA). Fluorescence microscopy was used to capture images of cofilin expression and localization in microglia. ImageJ software version 1.53t (NIH) was used to measure the fluorescence intensity of cofilin and microglia in the vicinity of the hemorrhagic region.

### Quantification of Microglial Morphology

Morphological alterations of microglia in mouse brain sections were analyzed using ImageJ software equipped with the FracLac and Skeletonize plugins as previously described [10, 13, 31]. Original images were processed into binary and outline photomicrographs before analysis. Convex hull and enclosing circle analyses were applied to determine cell span ratio, area, perimeter, and density. Fractal dimension (D_B_) was calculated using the box-counting method. Skeletonized images were used to quantify total branch length. All analyses were conducted on single-cell microglial outline images. For microglial morphological analysis, three independent animals per experimental group (n = 3) were analyzed. Multiple coronal sections containing the striatal region were examined for each animal, and three IBA1-positive microglial cells with complete and clearly distinguishable morphology were quantified per animal.

### Brain Tissue Collection and Morphometric Analysis

To explore potential genotype-associated differences in gross hemispheric dimensions and brain lateralization three days after ICH, we measured the maximal lengths of the right and left cerebral hemispheres and the anterior–posterior (A–P) brain dimension in male and female Flx, Het, and KO mice. Because these measurements were obtained during the acute post-ICH period, they may also reflect injury-associated swelling and were not intended to establish structural remodeling independently of edema. ICH was induced by collagenase injection into the left striatum; therefore, the left hemisphere was ipsilateral to the hematoma, and the right hemisphere was contralateral. Following euthanasia, whole brains were carefully extracted and rinsed in phosphate-buffered saline (PBS) to remove excess blood. Brains were imaged dorsally under standardized lighting and magnification conditions to allow morphometric quantification using a light microscope equipped with a Canon digital camera. As illustrated in panel (Supplementary Fig. 4 H), brain dimensions were assessed along three axes: the maximal length of the right hemisphere, the maximal length of the left hemisphere, and the anterior–posterior (A–P) axis. Measurements were performed manually using ImageJ (NIH), calibrated with a millimeter scale. Both hemispheres were measured independently to account for potential lateralization effects.

### Plasma Cofilin Measurement by ELISA

Plasma cofilin levels were quantified using a commercially available mouse cofilin ELISA kit (Biotechne, Minneapolis, MN, USA). For each assay, 10 μL of plasma was diluted with 90 μL of sample diluent and processed according to the manufacturer’s protocol to determine cofilin concentrations (pg/mL).

### Peripheral Leukocyte Quantification

Peripheral white blood cells, including neutrophils, lymphocytes, and monocytes, were quantified using the VETSCAN HM5 hematology analyzer (Abaxis, Union City, CA, USA). For this analysis, 100–200 µL of peripheral blood was collected into EDTA-coated tubes on day 3 and processed immediately on the instrument.

### Statistical Analysis

Data demonstrated a normal distribution, as confirmed by Shapiro–Wilk’s test. Statistical significance was defined as p < 0.05, and quantitative data were displayed as mean ± standard error of the mean (SEM). Comparisons between groups were performed using one-way or two-way analysis of variance (ANOVA), as appropriate, followed by Tukey’s multiple-comparisons test. Post hoc multiple-comparison tests were prespecified in the statistical analysis plan and were performed only when a significant main effect or interaction was identified by ANOVA. A two-tailed, unpaired Student’s t-test was used to compare the means of the two groups. All statistical analyses were conducted utilizing GraphPad Prism version 10.5 (GraphPad Software, San Diego, CA, USA). No animals were excluded from analysis. All surviving animals completed the planned behavioral and histological assessments.

## Results

### Generation and characterization of cofilin-1 knockout

To investigate the impact of cofilin-1 deletion in postnatal microglia, we crossed mice carrying the Cx3cr1Cre transgene, which expresses Cre specifically in microglia, with homozygous Cofilin^fl/fl^ (Flx) mice, which are flanked with loxP sites. As a result, we obtained the heterozygous strain CX3Cr1^Cre^.cofilin^fl/wt^, which was then crossed with Flx mice to yield the homozygous CX3Cr1^Cre^.cofilin^fl/fl^ KO mice (Supplementary Fig. 1 A). PCR genotyping confirmed the presence of the floxed, deleted, and wild-type alleles for cofilin-1 (470 bp, 170 bp, and 380 bp, respectively) and the Cx3cr1-Cre allele (302 bp) (Supplementary Fig. 1 B). Both male and female KO mice were viable and showed no gross morphological abnormalities compared to Flx controls (Supplementary Fig.  1 C).

To assess whether microglial cofilin-1 deletion affects overall growth or body condition, we measured body weight in male and female mice across the three genotypes (Flx, Het, KO). Both male and female Het and KO mice, matched in age, did not show significant changes in body weight after 8–10 weeks compared to Flx mice, Two-way ANOVA revealed no substantial genotype–sex interaction (F(2, 65) = 1.531, p = 0.2240) and no main effect of genotype (F(2, 65) = 0.8043, p = 0.4518). However, there was a strong main effect of sex (F(1, 65) = 42.63, p < 0.0001), with males weighing more than females regardless of genotype. The observed sexual dimorphism, in which males display higher body weight than females, is a well-documented phenomenon and is likely due to differences in hormonal regulation and metabolic processes between the sexes [32–34]. Post hoc comparisons showed no significant pairwise differences between genotypes in either males (Flx vs. Het, p = 0.2946; Flx vs. KO, p = 0.3572; Het vs. KO, p = 0.9670) or females (Flx vs. Het, p = 0.6239; Flx vs. KO, p = 0.9164; Het vs. KO, p = 0.3852) (Supplementary Fig. 2 A). These findings suggest that the specific deletion of cofilin-1 in microglia did not have a major impact on the overall growth and development of the mice.

**Fig. 2:**
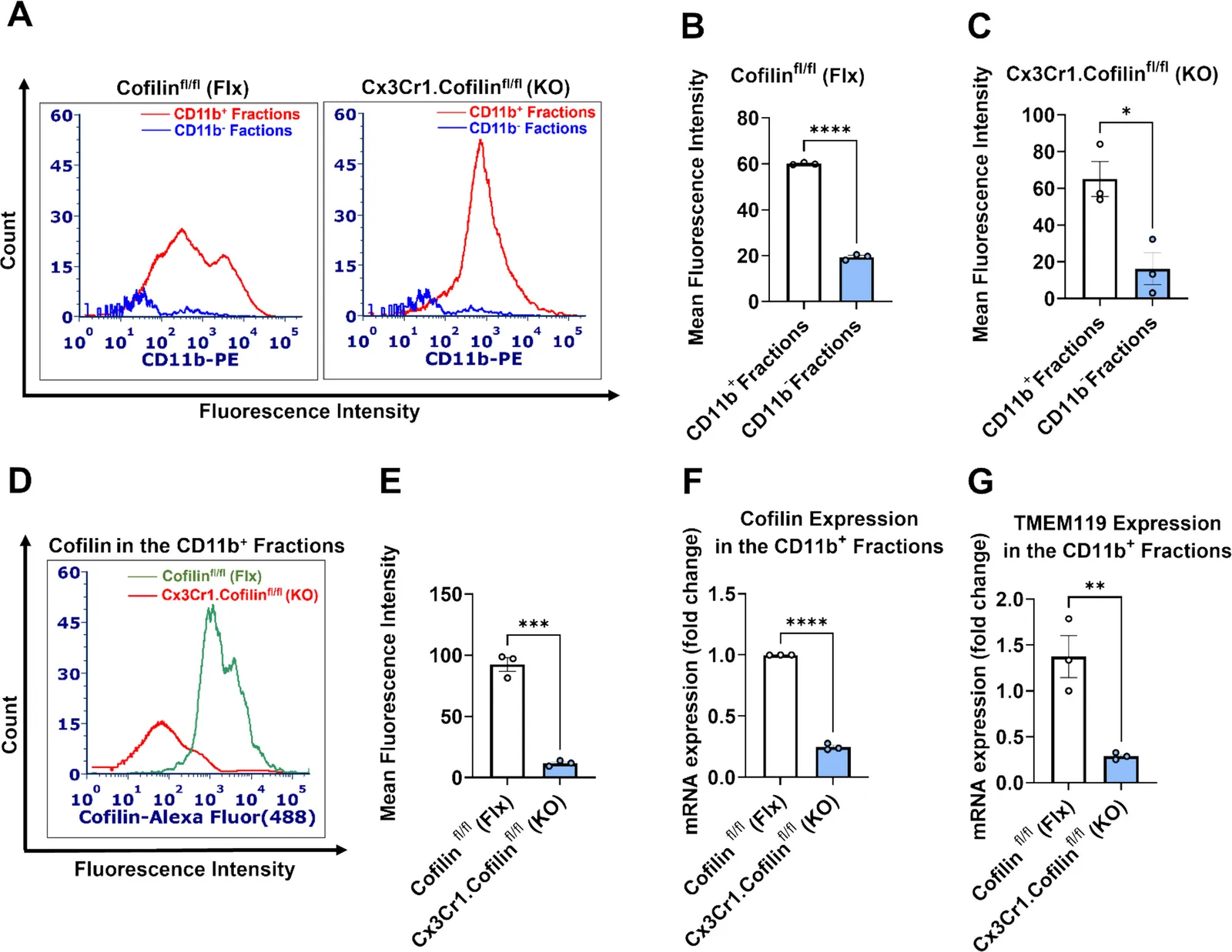
Flow cytometry and gene expression analysis of cofilin and TMEM119 in CD11b⁺ fraction from Cofilin^fl/fl^ (Flx) and CX3Cr1^Cre^.cofilin^fl/fl^ (KO) mouse brains. (**A**) Representative flow cytometry histograms showing CD11b⁺ (red) and CD11b⁻ (blue) cell populations in Flx and KO mice. (**B**–**C**) The bar graph represents the mean fluorescence intensity of both groups. It shows the quantification of mean fluorescence intensity for CD11b in CD11b⁺ and CD11b⁻ fractions in Flx (B) and KO (C) groups (*p = 0.019, ****p < 0.0001). (**D**) Flow cytometry analysis of cofilin-Alexa Fluor (488) in the CD11b^+^ Fractions (microglia) from Flx and KO mice. The green histogram shows green fluorescent protein expression in Flx mice. The red histogram represents the cells not labeled by Alexa Fluor (488) fluorescence in the KO mice. (**E**) The bar graph represents the mean fluorescence intensity (***p = 0.0001). (F–G) qPCR analysis of cofilin (**F**) and TMEM119 (**G**) mRNA expression in CD11b⁺ fractions, expressed as fold change (**p = 0.009, ****p < 0.0001). Data are expressed as mean ± SEM; ns indicates no significant difference, and p < 0.05 was considered significant. *Difference of both groups, n = 3, unpaired t-test

Next, we isolated microglia from Flx and KO mice using CD11b MicroBeads. Isolated microglia (positive fractions, CD11b^+^) and other cells (negative fractions, CD11b^−^) were then subjected to cell counting using flow cytometry. Both Flx and KO groups were sorted into CD11b^+^ and CD11b^−^ fractions. The histogram shows differences in CD11b expression between the CD11b^+^ and CD11b^−^ fractions. The results showed that more than 90% of isolated microglia were CD11b-PE positive. A rightward shift is noticeable in the population of CD11b + fractions, indicating an increased expression of PE fluorescent protein (Fig. 2A)**.** The bar graph shows the mean fluorescence intensity for CD11b + and CD11b- fractions in both groups. The results show that microglia levels significantly decreased in the negative fractions after isolation in Flx and KO groups (*p = 0.019 and ****p < 0.0001) **(**Fig. 2B–2C**).**

To confirm cofilin-1 deficiency in the microglia of KO mice, CD11b^+^ fractions (microglia) of Flx and KO mice were incubated with Alexa Fluor conjugated to cofilin-1. The green histogram shows a significant rightward shift in GFP expression in Flx mice. The red histogram represents the population not labeled by Alexa Fluor (488) fluorescence in the mutant mice **(**Fig. 2D**).** The results showed that cofilin-1 expression in CD11b + fractions (microglia) in mutant mice was significantly decreased compared with cofilin expression in CD11b^+^ fractions (microglia) in Flx mice (***p = 0.0001) **(**Fig. 2E**).** Moreover, qPCR analysis from sorted CD11b^+^ fractions (microglia) confirmed that the cofilin-1 gene was significantly diminished in the KO transgenic line (****p < 0.0001) **(**Fig. 2F**),** with minimal interference with systemic plasma cofilin-1 levels among all genotypes for both males and females. Two-way ANOVA revealed no significant genotype–sex interaction (F(2, 15) = 2.104, p = 0.1565) and no main effect of genotype (F(2, 15) = 0.06447, p = 0.9378). Notably, sex had a strong main effect (F(1, 15) = 18.34, p = 0.0007), with females having higher plasma cofilin levels than males. Post hoc analyses confirmed no significant pairwise differences between genotypes within each sex (males: Flx vs. Het, p = 0.7275; Flx vs. KO, p = 0.1978; Het vs. KO, p = 0.5589; females: Flx vs. Het, p = 0.9392; Flx vs. KO, p = 0.5217; Het vs. KO, p = 0.7243) (Supplementary Fig. 2 B).

**Fig. 3:**
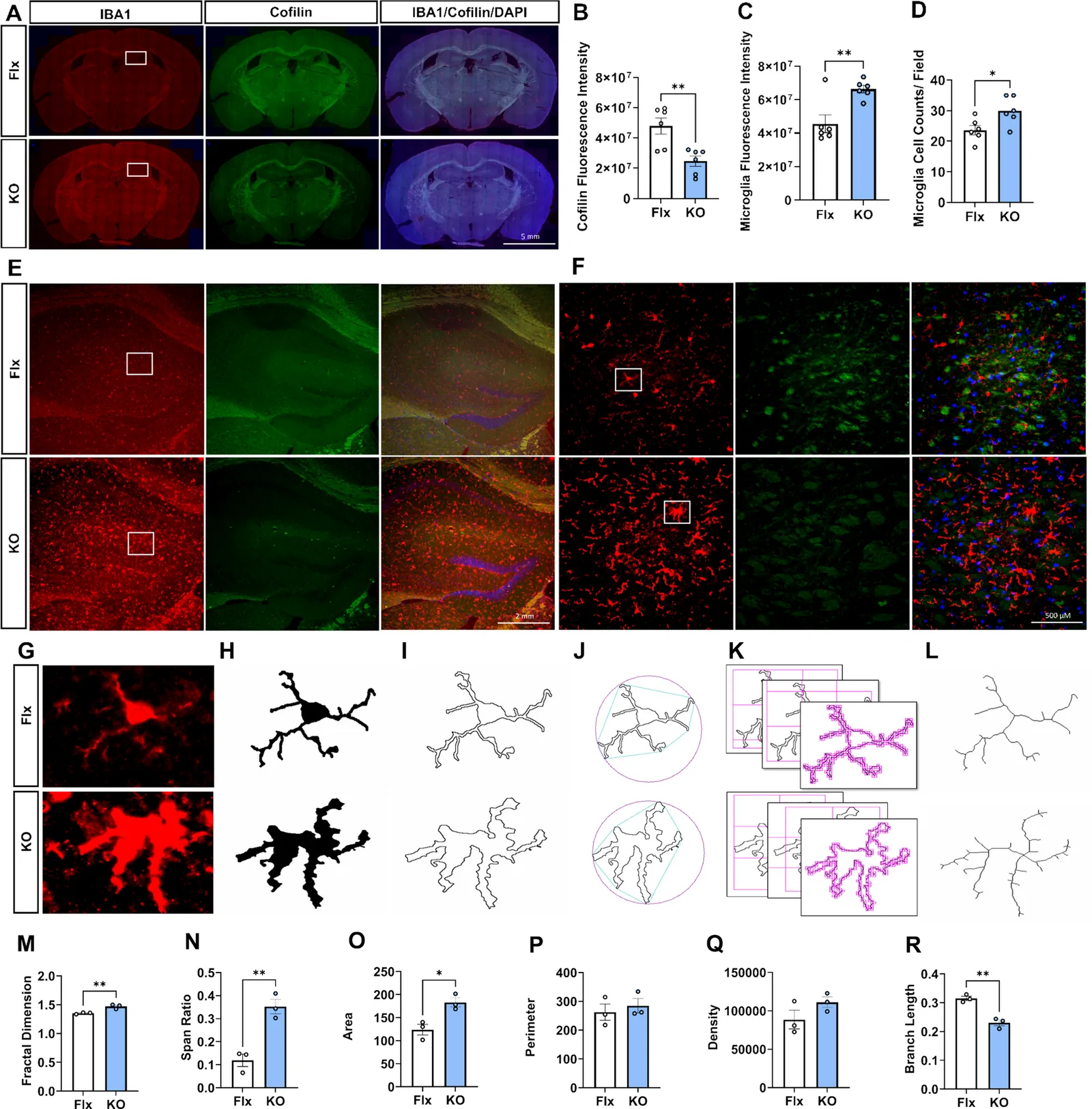
Immunohistochemical analysis of microglial activation and cofilin expression in Flx and KO mice. (**A**) Representative coronal brain sections from Flx and KO mice stained for IBA1 (microglial marker, red), cofilin (green), and DAPI (nuclear stain, blue). (**B**) Quantification of cofilin fluorescence intensity shows a significant reduction in KO compared to Flx mice (**p = 0.0044). (**C**) Quantification of IBA1 fluorescence intensity indicates increased microglial fluorescence intensity in KO mice (**p = 0.005). (**D**) Microglial cell counts per field, showing significantly higher numbers of microglia in KO mice (*p = 0.025). (**E–F**) Higher-magnification images of the hippocampal region showing IBA1 (microglial marker, red), cofilin (green), and DAPI (nuclear stain, blue), with KO brains displaying increased microglial density. (**G**) Enlarged images of individual microglia from Flx and KO mice. **(H–L)** Morphological analyses of microglia, including binary (**H**), outline (**I**), the convex hull (blue), and enclosing circle (pink) related to the corresponding outline shapes, are utilized to determine shape span ratio, area, perimeter, and density (**J**), an illustration of the box counting method is used for fractal dimension (D_B_) calculation (**K**), skeletonized microglia were used to calculate the branch length (**L**). (**M–R**) Quantitative assessment of microglial morphology demonstrated that KO microglia exhibited increased fractal dimension (**p = 0.0082) (**M**), span ratio (**p = 0.0048) **(N)**, and cell body area (*p = 0.0174) (**O**)**,** as well as reduced branch length (**p = 0.0027) (**R**), with no significant changes in perimeter (**P**) or density (**Q**). Data are presented as mean ± SEM, with individual data points shown. Six animals per group (n = 6), including male and female mice, were used for immunostaining. For microglial morphological analysis (panels M–R), three independent animals per group were analyzed, with three individual microglial cells assessed per animal. Statistical significance was determined using an unpaired two-tailed t-test, with p < 0.05 considered statistically significant. Scale bars: 5 mm (**A**), 2 mm (**E**), 500 μm (**F**)

Furthermore, analysis of the CD11b⁺ cell fraction revealed that TMEM119 expression, a marker of homeostatic microglia [35, 36], was significantly reduced in KO mice compared to Flx controls (**p = 0.009). Since previous studies have shown that TMEM119 is downregulated upon microglial activation [37, 38], this finding suggests a shift from a homeostatic to an activated phenotype in KO microglia **(**Fig. 2G**)**. Supporting this observation, immunohistochemical analysis showed markedly reduced cofilin immunoreactivity and increased microglial activation in KO brains compared to Flx controls **(**Fig. 3A, 3E and 3F**)**. Quantification revealed significantly reduced cofilin fluorescence intensity (**p = 0.0044) **(**Fig. 3B**)**, increased microglial fluorescence, and increased microglial counts/field, respectively (**p = 0.005, *p = 0.025) in KO mice **(**Fig. 3C–3D**)**, consistent with enhanced microglial activation and reduced cofilin expression.

To investigate the impact of cofilin-1 deficiency on microglial morphology, we used higher-magnification images focused on the hippocampal region. We observed a visibly increased density of microglia in KO brains compared with Flx controls **(**Fig. 3E–3F**)**. Enlarged views of individual microglia further emphasized distinct morphological alterations between groups **(**Fig. 3G**)**. Morphological features were quantified using multiple analytic approaches. Binary **(**Fig. 3H**)** and outline **(**Fig. 3I**)** representations of microglia, along with convex hull (blue) and enclosing circle (pink) analyses **(**Fig. 3J**)**, were used to find span ratio, area, perimeter, and density. The fractal dimension (DB) was calculated using the box-counting method **(**Fig. 3K**)**, and skeletonized images enabled the quantification of branch lengths **(**Fig. 3L**)**. Quantitative analysis demonstrated that KO microglia exhibited a significantly higher fractal dimension (DB; **p = 0.0082) **(**Fig. 3M**)**, increased span ratio (**p = 0.0048) **(**Fig. 3N**),** and enlarged cell body area (*p = 0.0174) **(**Fig. 3O**)**. However, branch length was markedly reduced in KO microglia (**p = 0.0027) **(**Fig. 3R**)**. No significant differences were observed in microglial perimeter or density **(**Figs. 3P–3Q**)**. Together, these findings suggest that cofilin-1 deficiency alters microglial morphology, leading to a more complex and activated state characterized by increased fractal dimension, soma hypertrophy, and reduced branching complexity.

### Neurobehavioral characterization of cofilin-1 knockout using rotarod, grip strength, inverted screen, wire hang, and open field tests

**Fig. 4:**
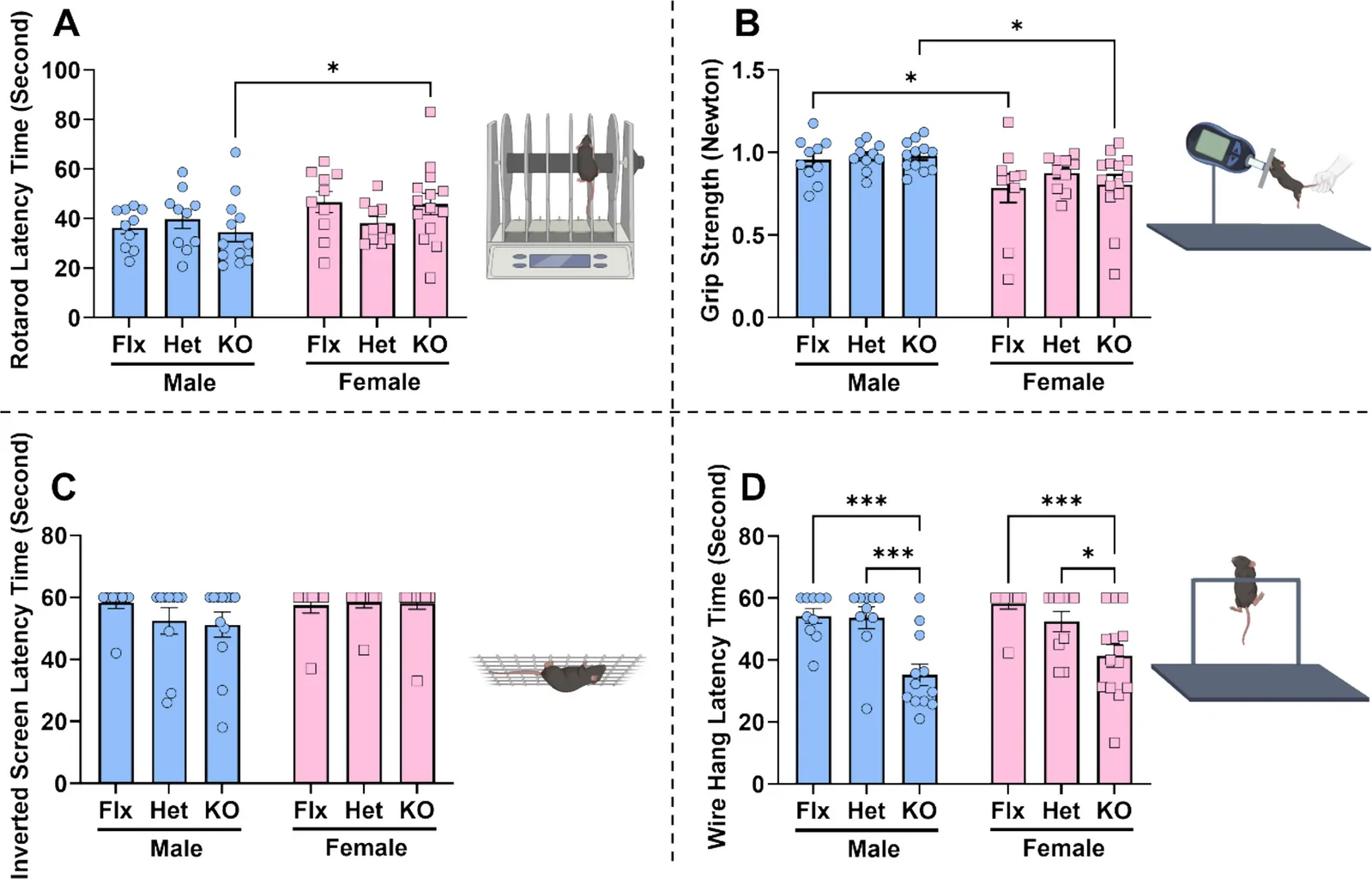
Motor coordination and neuromuscular strength in microglial cofilin-1 knockout (KO) mice. Motor performance and strength were evaluated in male and female mice of three genotypes (Flx, Het, KO) using four behavioral assays. **(A)** In the rotarod test, females exhibited longer latency to fall than males, but no genotype-dependent differences were observed. **(B)** Grip strength analysis revealed a significant main effect of sex, with males showing greater forelimb force than females, independent of genotype. **(C)** In the inverted screen test, performance was similar across all genotypes and sexes, indicating preserved neuromuscular endurance. **(D)** However, the wire hang test revealed a significant genotype effect, with KO mice of both sexes showing significantly shorter latency to fall than Flx and Het controls. Data are presented as mean ± SEM; each point represents an individual mouse. *p < 0.05, ***p < 0.001; two-way ANOVA with Tukey’s multiple comparisons test, n = 10 − 14

To evaluate whether cofilin-1 deletion in microglia affects motor coordination and balance, we performed an accelerating rotarod test in male and female mice of all genotypes (Flx, Het, KO). Two-way ANOVA showed no significant interaction between genotype and sex (F(2, 60) = 1.693, p = 0.1925) and no genotypic effect (F(2, 60) = 0.2129, p = 0.8089). However, sex had a significant main effect (F(1, 60) = 4.453, p = 0.039), with females having a slightly longer latency to fall than males, regardless of genotype. Post hoc pairwise comparisons showed no significant differences between genotypes in either sex (males: Flx vs. Het, p = 0.8220; Flx vs. KO, p = 0.9465; Het vs. KO, p = 0.6173; females: Flx vs. Het, p = 0.2888; Flx vs. KO, p = 0.9849; Het vs. KO, p = 0.3096). These findings indicate that microglial cofilin deletion does not impair motor coordination, although sex-related differences in performance were detected **(**Fig. 4A and Supplementary Table 3).

We also evaluated neuromuscular strength using grip strength, inverted screen, and wire hang tests (three training trials per day were performed for three consecutive days). First, grip strength was assessed to determine whether microglial cofilin deletion affects forelimb muscle performance in male and female mice across genotypes (Flx, Het, KO). Two-way ANOVA revealed no significant genotype–sex interaction (F(2, 60) = 0.3255, p = 0.7234) and no main effect of genotype (F(2, 60) = 0.5529, p = 0.5782). However, sex had a significant main effect (F(1, 60) = 12.82, p = 0.0007), with males showing higher grip strength than females regardless of genotype. Post hoc comparisons showed no significant differences between genotypes within each sex (males: Flx vs. Het, p = 0.9652; Flx vs. KO, p = 0.9491; Het vs. KO, p = 0.9990; females: Flx vs. Het, p = 0.4446; Flx vs. KO, p = 0.9441; Het vs. KO, p = 0.5813). These findings indicate that microglial cofilin deletion does not affect grip strength, although consistent sex-based differences in forelimb muscle performance were observed **(**Fig. 4B and Supplementary Table 3).

On the inverted screen test, which measures neuromuscular endurance and coordination, performance was comparable across genotypes and sexes. Two-way ANOVA showed no genotype–sex interaction (F(2, 59) = 0.9517, p = 0.3919), no genotype effect (F(2, 59) = 0.6297, p = 0.5363), and no sex effect (F(1, 59) = 2.749, p = 0.1026). Pairwise comparisons within each sex revealed no significant differences between Flx, Het, and KO groups (males: Flx vs. Het, p = 0.3777; Flx vs. KO, p = 0.2136; Het vs. KO, p = 0.9522; females: Flx vs. Het, p = 0.9797; Flx vs. KO, p = 0.9874; Het vs. KO, p = 0.9982) (Fig. 4C and Supplementary Table 3).

In contrast, the wire hang test revealed a clear genotype effect on muscle strength and fatigue resistance (F(2, 60) = 18.73, p < 0.0001). No sex difference (F(1, 60) = 1.246, p = 0.2688) or interaction (F(2, 60) = 0.6852, p = 0.5079) was detected. Post hoc tests indicated KO mice had significantly shorter hanging times compared to Flx (males: p = 0.0003; females: p = 0.0010) and Het (males: p = 0.0005; females: p = 0.0405) counterparts, whereas Flx and Het mice did not differ significantly (males: p = 0.9914; females: p = 0.4471) (Fig. 4D and Supplementary Table 3). Together with the grip strength data, these results indicate that microglial cofilin deletion does not affect forelimb force in the inverted screen task but selectively impairs wire hang performance, suggesting task-specific neuromuscular dysfunction.

**Fig. 5:**
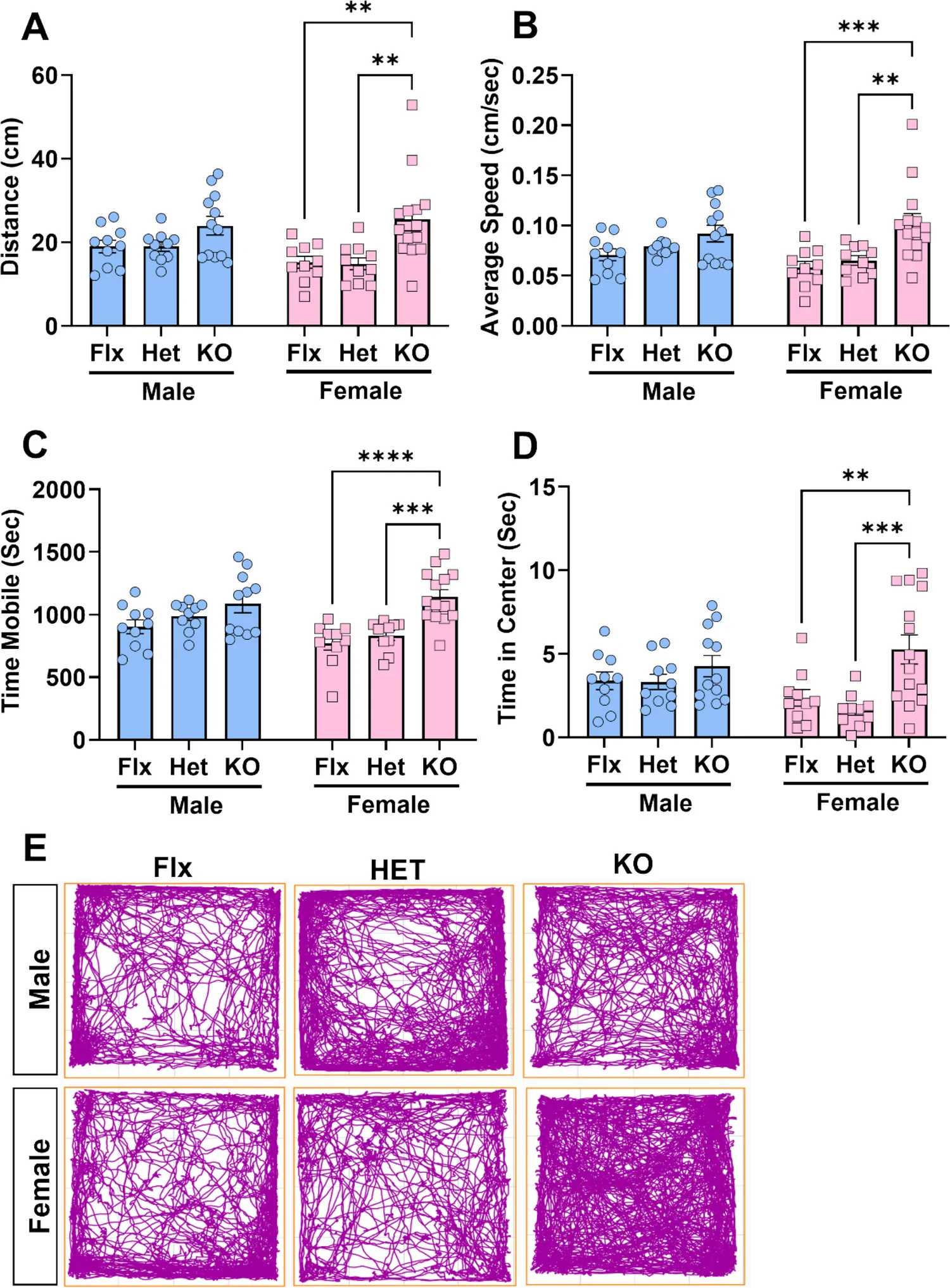
Open field assessment of locomotor activity and exploratory behavior in male and female cofilin-1 knockout (KO) mice. Open-field test outcomes are shown for **(A)** total distance traveled, **(B)** average speed, **(C)** total time mobile, and **(D)** time spent in the center of the arena, comparing Flx, Het, and KO mice in both males and females. **(E)** Representative movement tracking plots illustrate the locomotor patterns for each group. Data are presented as mean ± SEM; each point represents one animal. Statistical significance was determined by two-way ANOVA followed by Tukey’s multiple comparisons test (n = 10–14). **p < 0.01, ***p < 0.001, ****p < 0.0001

Next, on day 5, we performed the open-field test to determine if there were changes in locomotor activity and exploratory behavior in male and female mice of all genotypes (Flx, Het, KO). Animals were introduced to a novel open-field box and allowed to explore freely for 30 min. We evaluated four parameters in both sexes: total distance traveled, average speed, time mobile, and time spent in the central area. Distance traveled was significantly influenced by genotype (F(2, 60) = 10.18, p = 0.0002), with no significant genotype–sex interaction (F(2, 60) = 1.290, p = 0.2829) and no main effect of sex (F(1, 60) = 1.552, p = 0.2177). Post hoc comparisons showed no significant pairwise genotype differences in males (Flx vs. Het, p > 0.9999; Flx vs. KO, p = 0.2090; Het vs. KO, p = 0.2076). In females, KO mice traveled significantly more distance than both Flx (p = 0.0015) and Het (p = 0.0010) mice, with no difference between Flx and Het (p = 0.9926) (Figs. 5A and Supplementary Table 3). Average speed showed a similar pattern, with a significant main effect of genotype (F(2, 60) = 10.83, p < 0.0001), no genotype–sex interaction (F(2, 60) = 1.731, p = 0.1859), and no effect of sex (F(1, 60) = 0.8210, p = 0.3685). In males, speed did not differ significantly between genotypes (Flx vs. Het, p = 0.7256; Flx vs. KO, p = 0.1245; Het vs. KO, p = 0.4658). In females, KO mice exhibited significantly greater locomotor speed than both the Flx (p = 0.0002) and Het (p = 0.0021) groups, with no difference between the Flx and Het groups (p = 0.8041) (Figs. 5B and Supplementary Table 3). Time mobile was also significantly affected by genotype (F(2, 59) = 14.29, p < 0.0001), with no significant interaction (F(2, 59) = 2.348, p = 0.1044) and no effect of sex (F(1, 59) = 2.867, p = 0.0957). In males, there were no significant differences between genotypes (Flx vs. Het, p = 0.5376; Flx vs. KO, p = 0.0601; Het vs. KO, p = 0.4393). In females, KO mice spent more time mobile than Flx (p < 0.0001) and Het (p = 0.0003) mice, with no difference between Flx and Het (p = 0.7351) (Figs. 5C and Supplementary Table 3). Finally, time spent in the center, an index of anxiety-like behavior, was significantly influenced by genotype (F(2, 60) = 7.934, p = 0.0009) but not by sex (F(1, 60) = 1.201, p = 0.2776), with no significant interaction (F(2, 60) = 2.581, p = 0.0841). In males, there were no significant differences between genotypes (Flx vs. Het, p = 0.9972; Flx vs. KO, p = 0.5935; Het vs. KO, p = 0.5473). In females, KO mice spent significantly more time in the center compared to Flx (p = 0.0043) and Het (p = 0.0003) mice, with no difference between Flx and Het (p = 0.7107) (Figs. 5D and Supplementary Table 3). Overall, these findings suggest that microglial cofilin-1 deletion selectively enhances locomotor activity and exploratory behavior in KO females, exhibiting hyperactivity consistent with a hyperlocomotion phenotype, as well as decreased anxiety-related behavior **(**Figs. 5E**,** Illustrative movement tracking plots of all genotypes, including Flx, Het, and KO**).**

### Cofilin-1 knockout exhibits no altered behavioral responses in learning or working memory in novel object recognition or T-maze spontaneous alternation tests

To assess whether microglial cofilin-1 deletion influences recognition memory and spatial working memory, we conducted the novel object recognition test (NORT) and T-maze spontaneous alternation test in male and female mice across genotypes (Flx, Het, KO). For the NORT discrimination index, two-way ANOVA revealed no significant genotype–sex interaction (F(2, 60) = 0.3360, p = 0.7160), genotype effect (F(2, 60) = 0.1278, p = 0.8802), or sex effect (F(1, 60) = 0.5646, p = 0.4553). Post hoc analyses showed no differences between genotypes in either males (Flx vs. Het, p = 0.9368; Flx vs. KO, p = 0.9590; Het vs. KO, p = 0.8014) or females (Flx vs. Het, p = 0.7577; Flx vs. KO, p = 0.8980; Het vs. KO, p = 0.9431) (Supplementary Fig. 3 A and Supplementary Table 3). For the % total investigation time in NORT, there was similarly no significant interaction (F(2, 60) = 0.5071, p = 0.6048), genotype effect (F(2, 60) = 0.07452, p = 0.9283), or sex effect (F(1, 60) = 0.2450, p = 0.6224). No pairwise comparisons reached significance in males (Flx vs. Het, p = 0.9089; Flx vs. KO, p = 0.9946; Het vs. KO, p = 0.8550) or females (Flx vs. Het, p = 0.6371; Flx vs. KO, p = 0.9282; Het vs. KO, p = 0.8134) (Supplementary Fig. 3 B and Supplementary Table 3). In the T-maze spontaneous alternation task, two-way ANOVA showed no significant interaction (F(2, 61) = 0.3688, p = 0.6931), genotype effect (F(2, 61) = 1.485, p = 0.2345), or sex effect (F(1, 61) = 2.663, p = 0.1079). No genotype differences were detected in males (Flx vs. Het, p = 0.8302; Flx vs. KO, p = 0.8927; Het vs. KO, p = 0.9871) or females (Flx vs. Het, p = 0.1755; Flx vs. KO, p = 0.4031; Het vs. KO, p = 0.8046) (Supplementary Fig.  3 C and Supplementary Table 3). Overall, these results indicate that microglial cofilin-1 deletion does not impair recognition memory, exploratory preference, or spatial working memory in either sex.

### Cofilin-1 knockout impairs neurobehavioral function after ICH

ICH results in chronic motor and neurological impairments, and both genotype and sex can affect the degree of these impairments [10, 19, 39]. To study the role of cofilin-1 deficiency in microglia in ICH, three genotypes of male and female mice (Flx, Het, and KO) were subjected to collagenase-induced striatal ICH, and motor coordination, neuromuscular strength, and neurological outcomes were assessed on day 3 post-injury (experimental design shown in Fig. 1B). Two-way ANOVA was employed to determine the independent and interactional effects of sex and genotype, and subsequent post hoc tests were used to differentiate group-specific variation.

**Fig. 6:**
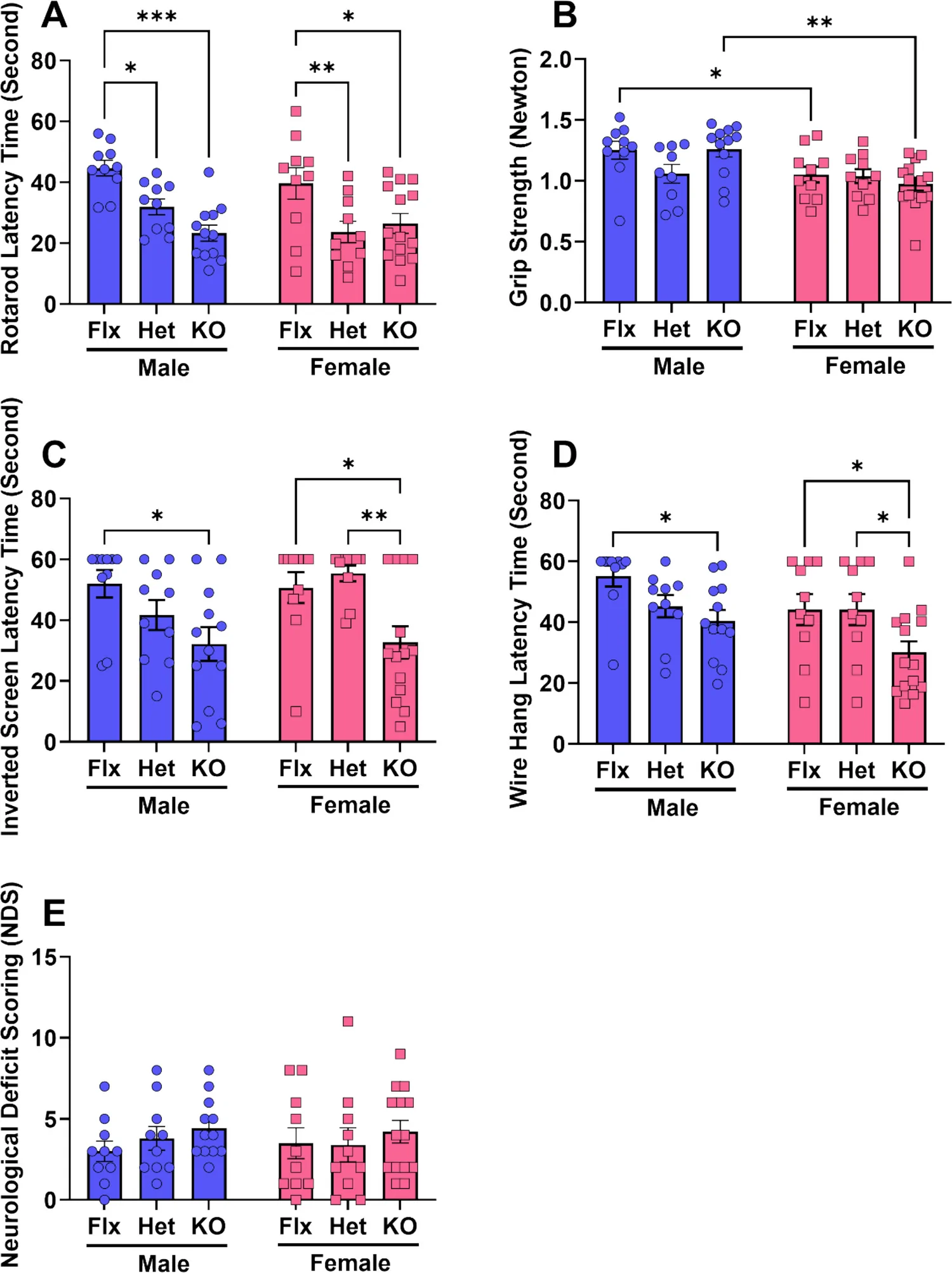
Motor coordination, strength, and neurological outcomes after ICH. Behavioral and motor performance were evaluated in male and female Flx, Het, and KO mice following ICH. (**A**) Rotarod latency to fall, assessing motor coordination and balance, revealed significant sex- and genotype-dependent differences, with reduced performance in KO mice. (**B**) Grip strength measurements indicated impaired forelimb strength, particularly in female KO mice. (**C**) Inverted screen latency exhibited deficits in neuromuscular endurance in male and female KO mice compared to Flx and Het mice. (**D**) Wire hang latency demonstrated both sex- and genotype-dependent impairments, consistent with reduced neuromuscular strength and coordination. (**E**) NDS revealed no significant genotype-dependent effects, though a trend toward greater deficits was observed in females. Data are presented as mean ± SEM, with individual values shown. Statistical analyses were performed using two-way ANOVA followed by Tukey’s multiple comparisons test. *p < 0.05, **p < 0.01, ***p < 0.001 (n = 10 − 14)

Latency to fall on accelerated rotarod using two-way ANOVA revealed a significant effect of genotype (F(2, 60) = 14.58, P < 0.0001), while neither sex (F(1, 60) = 1.473, P = 0.2297) nor the interaction between sex and genotype (F(2, 60) = 1.610, P = 0.2085) reached significance. Post hoc comparisons showed that in males, Flx mice performed significantly better than Het (P = 0.0353) and KO mice (P = 0.0001), whereas Het and KO did not differ (P = 0.1791). In females, Flx also outperformed both Het (P = 0.0065) and KO (P = 0.0169), while Het and KO were comparable (P = 0.8203). These findings establish a strong genotype-dependent impairment in motor coordination that is independent of sex **(**Fig. 6A and Supplementary Table 4). Next, analysis of grip strength revealed a significant effect of sex (F(1, 59) = 10.24, P = 0.0022), with females overall displaying reduced forelimb strength compared with males. No significant effect of genotype (F(2, 59) = 1.211, P = 0.3053) or genotype – sex interaction (F(2, 59) = 2.236, P = 0.1159) was observed. Post hoc tests confirmed no significant pairwise differences among genotypes within either sex (all P > 0.05). Thus, grip strength impairment after ICH is predominantly sex-dependent (Fig. 6B and Supplementary Table 4).

For inverted screen, two-way ANOVA identified a significant effect of genotype (F(2, 60) = 9.182, P = 0.0003), but not sex (F(1, 60) = 1.134, P = 0.2911) or their interaction (F(2, 60) = 1.291, P = 0.2825). In males, Flx mice outperformed KO mice (P = 0.0168), whereas Flx vs. Het (P = 0.3417) and Het vs. KO (P = 0.3660) comparisons were not significant. In females, both Flx vs. KO (P = 0.0266) and Het vs. KO (P = 0.0039) were significant, indicating a genotype-dependent impairment in female KO mice **(**Fig. 6C and Supplementary Table 4). In addition, analysis of wire hang latency showed significant main effects of genotype (F(2, 60) = 6.678, P = 0.0024) and sex (F(1, 60) = 4.865, P = 0.0313), but no interaction (F(2, 60) = 0.8592, P = 0.4287). In males, KO mice exhibited reduced latency compared with Flx (P = 0.0375), while Flx vs. Het (P = 0.2427) and Het vs. KO (P = 0.6894) were not significant. In females, KO mice performed worse than both Flx (P = 0.0399) and Het (P = 0.0399), while Flx and Het were indistinguishable (P > 0.9999). These data highlight sex-dependent vulnerability, with females showing a broader genotype effect (Fig. 6D and Supplementary Table 4). Moreover, neurological deficit score (NDS) using two-way ANOVA revealed no significant effects of genotype (F(2, 60) = 1.047, P = 0.3573), sex (F(1, 60) = 0.0029, P = 0.9573), or interaction (F(2, 60) = 0.1742, P = 0.8405). Pairwise comparisons confirmed no significant differences among groups (all P > 0.40). Thus, while motor coordination and neuromuscular strength exhibited sex- and genotype-specific deficits, global neurological scoring was not sensitive to these distinctions (Fig. 6E and Supplementary Table 4).

**Fig. 7:**
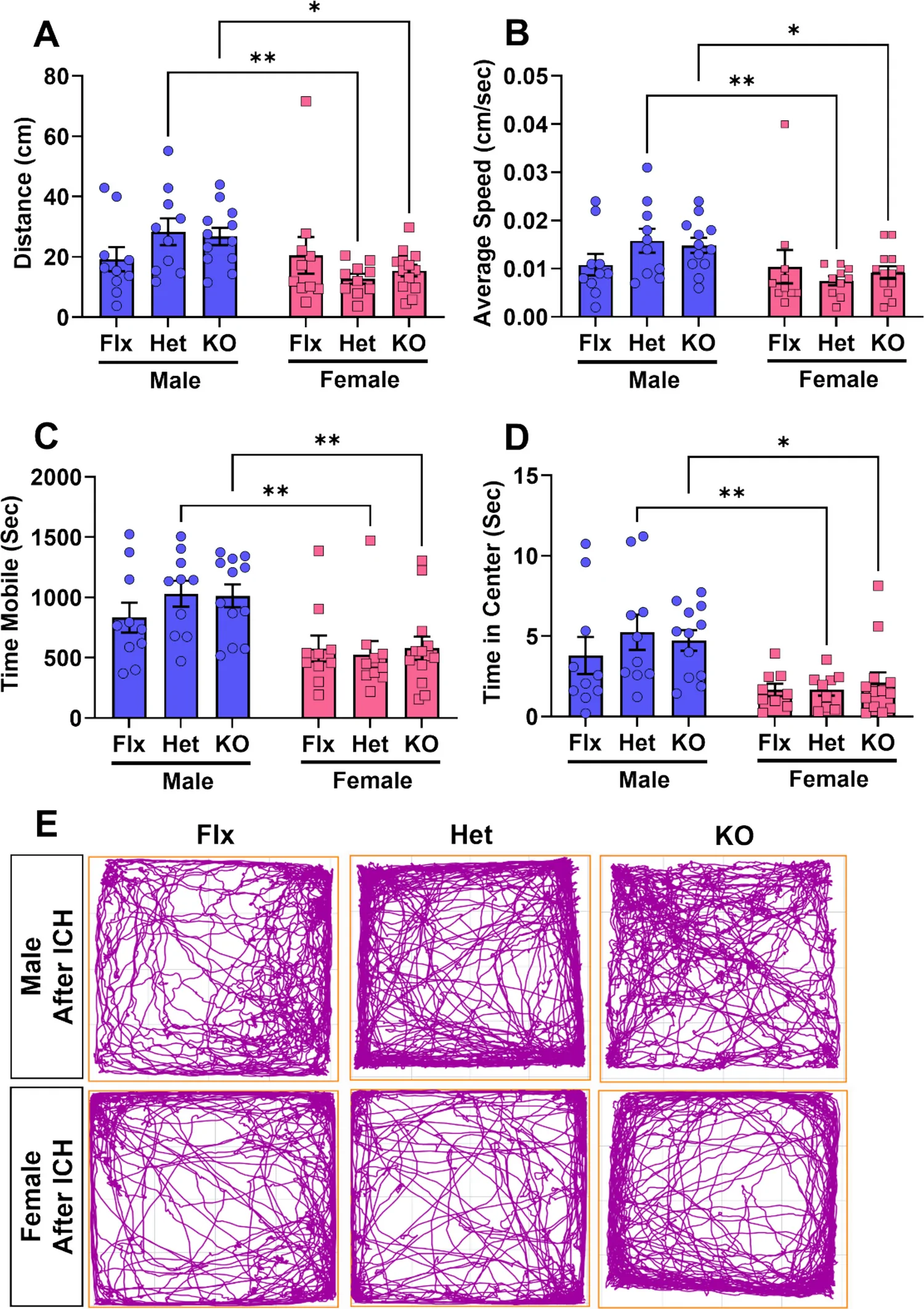
Open field performance following ICH in male and female Flx, Het, and KO mice. (**A**) Total distance traveled, (**B**) average speed, (**C**) time spent mobile, and (**D**) time spent in the center of the arena. Two-way ANOVA revealed a significant main effect of sex, but not genotype, across all parameters, with females consistently exhibiting reduced locomotor and exploratory activity compared to males. Post hoc comparisons confirmed that there were no significant genotype-dependent differences within each sex group. (**E**) Representative movement traces from male and female mice of each genotype after ICH. Data are presented as mean ± SEM, with individual values shown. *p < 0.05, **p < 0.01, (n = 10–14)

To assess locomotor and exploratory activity following ICH, we conducted the open-field test. For total distance traveled, there was no significant interaction between genotype and sex (F(2,59) = 2.744, P = 0.0726) and no main effect of genotype (F(2,59) = 0.0615, P = 0.9404). In contrast, sex exerted a significant impact (F(1,59) = 8.194, P = 0.0058). Post hoc pairwise comparisons revealed no significant differences among genotypes in males (Flx vs. Het: P = 0.2089; Flx vs. KO: P = 0.3070; Het vs. KO: P = 0.9493) or females (Flx vs. Het: P = 0.3202; Flx vs. KO: P = 0.5749; Het vs. KO: P = 0.8527) **(**Fig. 7A and Supplementary Table 4). Similarly, for average speed, no genotype–sex interaction was observed (F(2,59) = 1.750, P = 0.1827), and genotype was not significant (F(2,59) = 0.2556, P = 0.7753). However, a significant effect of sex was observed (F(1,59) = 7.885, P = 0.0067). Post hoc testing indicated no differences between genotypes in males (Flx vs. Het: P = 0.2359; Flx vs. KO: P = 0.3556; Het vs. KO: P = 0.9412) or in females (Flx vs. Het: P = 0.6093; Flx vs. KO: P = 0.9123; Het vs. KO: P = 0.8181) (Fig. 7B and Supplementary Table 4). Analysis of time spent mobile, neither the genotype–sex interaction (F(2,59) = 0.6859, P = 0.5076) nor genotype (F(2,59) = 0.4149, P = 0.6623) reached significance, whereas sex showed a robust main effect (F(1,59) = 20.92, P < 0.0001). Post hoc analyses confirmed no differences among genotypes in males (Flx vs. Het: P = 0.4139; Flx vs. KO: P = 0.4494; Het vs. KO: P = 0.9923) or females (Flx vs. Het: P = 0.9447; Flx vs. KO: P > 0.9999; Het vs. KO: P = 0.9332) (Fig. 7C and Supplementary Table 4). Last, for time spent in the center of the arena, which reflects anxiety-like behavior, no significant interaction was found (F(2,57) = 0.3967, P = 0.6744), and genotype was not significant (F(2,57) = 0.5231, P = 0.5955). By contrast, sex was highly significant (F(1,57) = 18.58, P < 0.0001). Post hoc comparisons showed no significant genotype differences in males (Flx vs. Het: P = 0.4122; Flx vs. KO: P = 0.6677; Het vs. KO: P = 0.8822) or females (Flx vs. Het: P = 0.9999; Flx vs. KO: P = 0.9090; Het vs. KO: P = 0.9150) (Fig. 7D and Supplementary Table 4). Representative movement lines illustrate reduced overall distance and exploration in females compared with males **(**Fig. 7E**)**. Taken together, these findings demonstrate that open field behavior after ICH is strongly influenced by sex but not by genotype, with females exhibiting altered locomotor and exploratory activity compared to males.

### Effect of cofilin-1 knockout on brain edema and hematoma volume after ICH

**Fig. 8:**
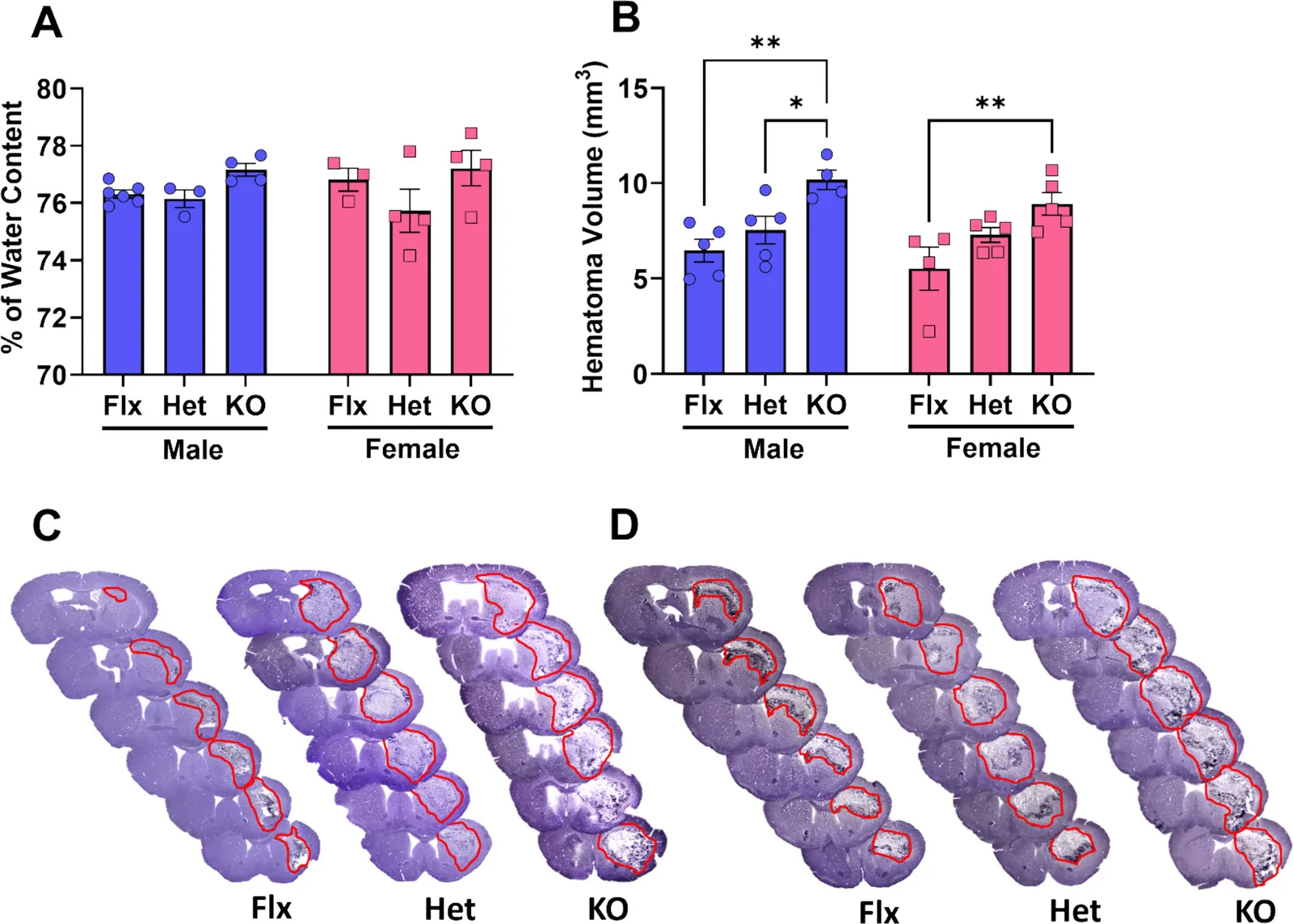
Brain water content and hematoma volume on day 3 after ICH in male and female Flx, Het, and KO mice. (**A**) Percentage of water content in the ipsilateral hemisphere, measured using the wet–dry method, revealed a main effect of genotype but no significant sex differences. (**B**) Quantification of hematoma volume demonstrated significantly larger lesions in KO mice compared with Flx and Het controls in both sexes. Data are mean ± SEM; individual points represent single animals. Statistical significance was assessed using two-way ANOVA followed by Tukey’s multiple comparisons test. (**C–D**) Representative cresyl violet–stained coronal brain sections from male (**C**) and female (**D**) mice, with hematoma boundaries outlined in red, illustrate the differences in lesion size among genotypes (Flx, Het, and KO). Data are presented as mean ± SEM, with individual data points representing the results from single animals. Statistical analyses were performed using two-way ANOVA followed by Tukey’s multiple comparisons test. *p < 0.05, **p < 0.01, (n = 3–6)

To evaluate post-injury brain edema, we measured the percentage of brain water content three days after ICH using the wet–dry weight method. This assay provides a direct quantification of tissue swelling and is widely used to assess the severity of edema following brain injury. Two-way ANOVA reached only borderline significance for genotype (F(2,18) = 3.663, p = 0.0463), but no significant interaction between genotype and sex (F(2,18) = 0.4920, p = 0.6194) and no main effect of sex (F(1,18) = 0.01481, p = 0.9045) were observed. Post hoc comparisons indicated no significant differences among genotypes in males (Flx vs. Het, p = 0.9617; Flx vs. KO, p = 0.3262; Het vs. KO, p = 0.3177) or females (Flx vs. Het, p = 0.2720; Flx vs. KO, p = 0.8251; Het vs. KO, p = 0.0717) **(**Fig. 8A**).**

We next quantified hematoma volume to assess lesion size and hematoma resolution after ICH. Two-way ANOVA identified a notable main effect of genotype (F(2,22) = 13.56, p = 0.0001), with no significant interaction (F(2,22) = 0.2976, p = 0.7455) and no main effect of sex (F(1,22) = 2.196, p = 0.1526). Post hoc analysis showed that in males, KO mice had significantly larger hematomas compared with both Flx (p = 0.0026) and Het (p = 0.0330) mice, with no difference between Flx and Het (p = 0.4786). In females, KO mice also displayed larger hematomas than Flx (p = 0.0056), whereas no significant differences were found between Flx and Het (p = 0.1885) or Het and KO (p = 0.1995) **(**Fig. 8B–8D**)**. These findings indicate that microglial cofilin-1 deficiency exacerbates hemorrhagic lesion size independent of sex.

### Cofilin-1 knockout modulates cofilin expression and microglial activation following ICH

**Fig. 9:**
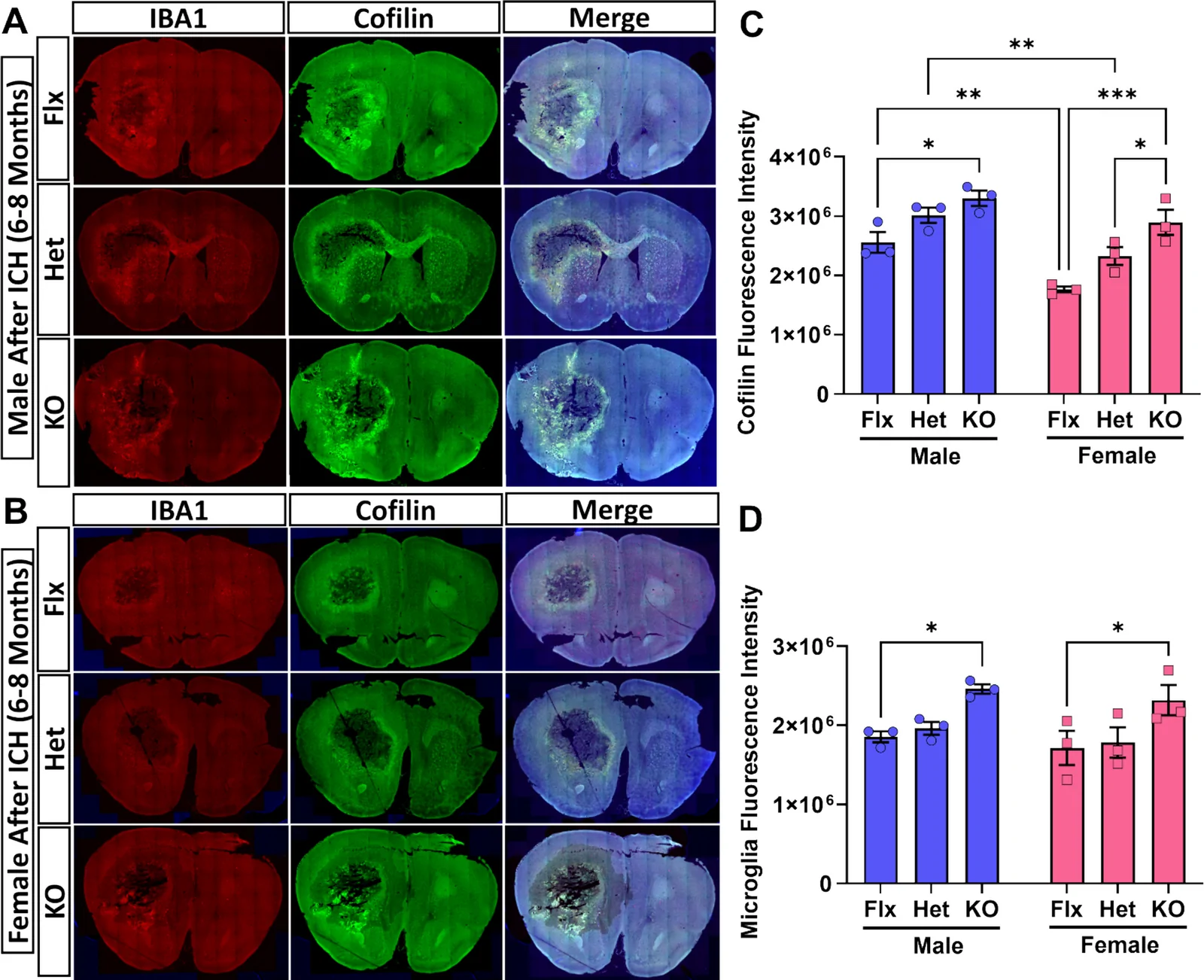
Cofilin-1 deficiency enhances microglial activation in a sex-dependent manner following ICH (n = 3). (A–B) Representative immunofluorescence images of coronal brain sections from male **(A)** and female **(B)** mice after ICH, showing IBA1 (red, microglial marker), cofilin-1 (green), and merged channels with DAPI (blue, nucleus). Genotypes include Flx, Het, and KO mice. **(C)** Quantitative analysis of cofilin-1 fluorescence intensity demonstrated a significant increase in KO compared with Flx and Het groups, with more effects observed in females (total cofilin-1 immunoreactivity represents all cell types within the field and is not restricted to microglia). **(D)** Quantification of IBA1 fluorescence intensity indicated elevated microglial activation in KO mice relative to Flx, with sex-specific differences. Data are expressed as mean ± SEM; statistical significance was assessed by two-way ANOVA followed by Tukey’s multiple comparisons test (*p < 0.05, **p < 0.01, *** p < 0.001)

To evaluate whether microglial cofilin-1 deletion alters cofilin expression and microglial activation following ICH in the ipsilateral region (around the hematoma), we performed immunohistochemistry on coronal brain sections stained with IBA1 (microglia marker, red) and cofilin (green). Fluorescence intensities were quantified to assess changes across sex and genotype. Two-way ANOVA revealed that cofilin fluorescence intensity was significantly affected by both genotype (F(2,12) = 19.67, P = 0.0002) and sex (F(1,12) = 26.46, P = 0.0002), whereas their interaction was not significant (F(2,12) = 0.8875, P = 0.4371). Post hoc analysis showed that in males, cofilin expression was significantly increased in KO compared with Flx mice (P = 0.0109), while Het mice were intermediate. In females, KO mice exhibited markedly increased cofilin expression relative to both Flx (P = 0.0005) and Het (P = 0.0490), suggesting a more pronounced genotype effect in females **(**Figs. 9A, 9B and 9C**)**. For microglial IBA1 intensity, two-way ANOVA demonstrated a main effect of genotype (F(2,12) = 9.516, P = 0.0033) but not sex (F(1,12) = 1.560, P = 0.2355), with no significant interaction (F(2,12) = 0.01006, P = 0.9900). Post hoc comparisons showed higher IBA1 intensity in KO mice than in Flx in both males (P = 0.0357) and females (P = 0.0360), while Het mice again showed intermediate levels. These results indicate that cofilin-1 deletion enhances microglial activation after ICH, with sex-dependent differences most evident in cofilin expression but not in IBA1 intensity **(**Figs. 9A, 9B, and 9D**)**.

### Cofilin-1 deficiency does not alter overall brain morphology but reveals subtle hemisphere-specific differences after ICH

To explore whether cofilin-1 genotype was associated with differences in gross hemispheric dimensions and potential brain lateralization during the acute post-ICH period, we measured right and left hemispheric lengths and the anterior–posterior (A–P) dimension in male and female Flx, Het, and KO mice. The left striatum was the site of collagenase injection; therefore, the left hemisphere was ipsilateral to the hematoma. These measurements were interpreted as exploratory morphometric observations, recognizing that post-injury swelling may influence brain dimensions. The existing measurements were compared across genotypes and sexes (Supplementary Figs. 4 A–H). For female mice, two-way ANOVA revealed a significant main effect of genotype (*F*(2,24) = 3.586, *p* = 0.0434) and right and left hemisphere length (*F*(1,24) = 163.0, *p* < 0.0001), but no interaction (*F*(2,24) = 0.062, *p* = 0.9400). Post hoc comparisons indicated no significant pairwise differences in either right or left hemispheric length among Flx, Het, or KO groups (all *p* > 0.1; Supplementary Fig. 4 A). In male mice, a significant main effect of genotype was detected (*F*(2,24) = 5.257, *p* = 0.0128), along with a remarkable right and left hemisphere length effect (*F*(1,24) = 72.73, *p* < 0.0001), but without interaction (*F*(2,24) = 0.678, *p* = 0.5171). Post hoc testing showed that the left hemisphere of KO mice was longer than that of Het mice (*p* = 0.0300), whereas right hemisphere lengths did not differ significantly between genotypes (all *p* > 0.1; Supplementary Fig. 4 A).

When male and female hemispheres were directly compared, a significant main effect of genotype (*F*(2,24) = 3.910, *p* = 0.0339) and sex (*F*(1,24) = 6.925, *p* = 0.0146) was observed for right hemisphere length, with no interaction (*F*(2,24) = 0.104, *p* = 0.9016). However, post hoc tests showed no pairwise differences within sex groups **(**all *p* > 0.1; Supplementary Fig.  4 C). For left hemispheric length, genotype also had a significant effect (*F*(2,24) = 5.464, *p* = 0.0111), while sex and interaction were not significant. Pairwise testing revealed a reduction in KO compared with Het males (*p* = 0.0125), but no significant differences in females (all *p* > 0.28; Supplementary Fig. 4D).

Lastly, analysis of A–P axis length revealed no significant effects of genotype (*F*(2,24) = 2.037, *p* = 0.1524), sex (*F*(1,24) = 2.624, *p* = 0.1183), or their interaction (*F*(2,24) = 0.209, *p* = 0.8132; Fig. E). Post hoc tests confirmed no differences between Flx, Het, or KO in either sex (all *p* > 0.3; Supplementary Fig. 4E). Together, these findings demonstrate that global brain size along the A–P axis is unaffected by cofilin-1 deletion, but subtle, genotype-dependent reductions in hemispheric length are present, particularly in the left hemisphere of male KO mice.

### Cofilin Deficiency Differentially Shapes Peripheral Monocyte, Lymphocyte, and Neutrophil Responses After ICH

To evaluate the systemic inflammatory response following ICH, we quantified peripheral leukocyte populations (monocytes, lymphocytes, and neutrophils) and plasma cofilin levels in male and female mice across genotypes (Flx, Het, KO). A two-way ANOVA was applied to determine the independent and interactive effects of sex and genotype. Analysis of monocytes revealed no significant interaction between sex and genotype (*F*(2,29) = 1.045, *P* = 0.3645), and sex alone did not influence monocyte counts (*F*(1,29) = 0.613, *P* = 0.4401). However, genotype exerted a significant effect (*F*(2,29) = 5.068, *P* = 0.0130). Post hoc testing revealed no significant differences among the Flx, Het, and KO groups in males (all *P* > 0.3). However, in females, KO animals displayed significantly increased monocyte counts compared to Flx (*P* = 0.0124) and Het (*P* = 0.0203) controls, while Flx vs. Het remained comparable (*P* = 0.9751) (Supplementary Fig. 5 A).

For lymphocytes, no sex—genotype interaction was detected (*F*(2,29) = 0.633, *P* = 0.5383). Both genotype (*F*(2,29) = 8.427, *P* = 0.0013) and sex (*F*(1,29) = 7.207, *P* = 0.0119) significantly affect lymphocyte counts. In males, no significant pairwise differences were found (all *P* > 0.1). In females, KO mice exhibited markedly higher lymphocyte levels compared to Flx (*P* = 0.0021); however, Het vs. KO was not significant (*P* = 0.0829) (Supplementary Fig. 5 B).

Next, neutrophil analysis demonstrated no significant interaction (*F*(2,29) = 0.183, *P* = 0.8334), but both genotype (*F*(2,29) = 12.07, *P* = 0.0002) and sex (*F*(1,29) = 7.029, *P* = 0.0129) were significant main factors. In males, KO animals showed significantly higher neutrophil counts compared with both Flx (*P* = 0.0083) and Het (*P* = 0.0116), while Flx vs. Het remained unchanged (*P* = 0.9896). Similarly, in females, KO mice had significantly increased neutrophils compared with Flx (*P* = 0.0117) and Het (*P* = 0.0364), with no difference observed between Flx vs. Het (*P* = 0.872) (Supplementary Fig.  5 C).

ELISA measurements of plasma cofilin revealed no significant sex—genotype interaction (*F*(2,21) = 0.990, *P* = 0.388), nor main effects of genotype (*F*(2,21) = 1.736, *P* = 0.201) or sex (*F*(1,21) = 1.930, *P* = 0.179). Post hoc analyses confirmed no significant differences across groups in either males or females (all *P* > 0.2) **(Supplementary Fig. 5D)**. Collectively, these results demonstrate that cofilin deficiency significantly modulates the peripheral immune response to ICH, resulting in sex- and genotype-dependent reductions in the populations of monocytes, lymphocytes, and neutrophils. In contrast, no change in plasma cofilin concentrations is observed.

## Discussion

The present study provides the first comprehensive characterization of microglial-specific cofilin-1 deficiency using a CX3Cr1-Cre-driven conditional knockout model under homeostatic conditions and after collagenase-induced ICH in both male and female mice. Our results demonstrate that deficiency of cofilin-1 in microglia disrupts basal microglial homeostasis, changes morphology in a manner suggestive of disturbed process dynamics, and induces refined sex-dependent behavioral alterations without broadly compromising baseline motor or cognitive function. Critically, in the context of ICH, microglial cofilin-1-deficient mice in the present study show aggravated secondary brain injury, increased hematoma volume, enhanced microglial activation, exacerbated neurobehavioral deficits, and modulated peripheral inflammatory responses. Core pathological findings related to lesion size were unaffected by sex; however, the effects on behavioral and immune outcomes were sex specific. Collectively, these results identified cofilin-1 as a pivotal regulator of microglial actin cytoskeleton dynamics, with its chronic absence from birth shifting microglia toward a maladaptive state that amplifies ICH-induced damage, in contrast to the neuroprotective effects observed with acute pharmacological inhibition [13] or siRNA-mediated knockdown after ICH [12].

Cofilin-1 is an essential actin-binding protein that facilitates the severing and depolymerization of F-actin, enabling the rapid turnover required for microglial process extension, retraction, and surveillance [6, 7]. Validation experiments confirmed the robust deletion of cofilin-1 in CD11b^+^ microglia using flow cytometry, qPCR, and immunohistochemistry, with > 90% purity in isolated cells and a decrease in cofilin-1 expression in the immunohistochemistry of KO brains. At homeostasis, KO microglia showed marked morphological changes, including increased fractal dimension, span ratio, and lacunarity, while simultaneously decreasing endpoints and process length. These changes exhibited a more compact and less ramified morphology, with disrupted branching complexity, consistent with a previous study that demonstrated impaired actin treadmilling and reduced motility [40]. Notably, TMEM119 mRNA expression, a homeostatic microglial marker, was substantially decreased in KO microglia, indicating destabilization of microglial identity and potential priming toward reactive states [38]. Our finding that microglial cofilin-1 KO reduces TMEM119 expression and primes microglia, and further exacerbates activation after ICH, is consistent with recent studies. For example, a decrease in TMEM119 in microglia, either with genetic KO [41, 42] or pathological downregulation (e.g., traumatic brain injury [43]), suggests the induction of a primed microglial state characterized by morphological activation (amoeboid shift, reduced branching) and impaired homeostasis, which is in parallel with our observed microglia hyperactivation at baseline and later worsened ICH outcomes. Although the present findings demonstrate altered microglial morphology, classical M1/M2 polarization markers were not evaluated. Future studies incorporating phenotype-specific molecular markers will further define the activation states associated with chronic microglial cofilin-1 deficiency. Furthermore, our novel KO model reveals baseline perturbations without overt neurodegeneration, which differs from the neuronal-specific cofilin-1/ADF double knockout, which induces severe synaptic defects and cognitive impairment [16]. The lack of cofilin-1 in microglia at baseline further distinguishes chronic genetic loss in our model from previous studies of acute oxidative stress-induced cofilin-actin rods following ICH [8–10]. Thus, cofilin-1 in microglia at baseline appears to be essential for maintaining a ramified surveillance morphology, and its deficiency may compromise barrier functions or debris clearance even before an insult occurs.

At baseline, neurobehavioral tests revealed no genotype effects on motor coordination (rotarod), grip strength, inverted screen, spatial working memory (T-maze), or recognition memory (NORT). These results suggest that microglial cofilin-1 loss does not affect the cognitive deficits observed in neuronal KO models [14, 15] or the ADHD-like hyperactivity in ADF/cofilin double mutants [17]. In contrast, the wire hang test showed impaired neuromuscular performance in both male and female mice. Moreover, open-field testing revealed sex-specific changes: female KO mice were hyperlocomotor and showed reduced anxiety-like behavior (increased center time), while males did not demonstrate any changes. These results suggest an interaction between cofilin-1 deficiency and sex hormones in modulating exploratory drive, via estrogen-mediated actin signaling [32]. Female-specific hyperlocomotion may reflect compensatory mechanisms or disrupted inhibitory circuits, echoing the sex-dimorphic microglial pruning that occurs during development [44]. Overall, baseline neurobehavioral integrity despite morphological disruptions underlines microglial resilience and points toward vulnerability in females under stress.

Following ICH, KO mice exhibited significantly larger hematoma volumes compared to Flx and Het controls, independent of sex. These pathological changes were associated with worsened motor deficits (reduced rotarod latency, inverted screen time, and wire-hang scores) after 3 days of ICH. Additionally, Grip strength differed between sexes, whereas no significant effect of genotype was observed. Microglial activation, assessed by IBA1 positive density and morphology near the hematoma, was significantly increased in KO mice, with intermediate effects in Het mice. An unexpected finding was that total cofilin-1 immunofluorescence in the perilesional region was increased, not decreased, in KO mice compared with Flx mice after ICH (Fig. 9), despite efficient cofilin-1 deletion in microglia at baseline (Figs. 2–3). We consider three non-exclusive explanations. First, compensatory upregulation of cofilin-1 in non-microglial, Cre-negative cell types (neurons, astrocytes, or endothelial cells) responding to greater local tissue injury and larger hematomas in KO mice could elevate the bulk signal even though microglial cofilin-1 itself remains deleted. Second, because CX3CR1 is expressed on circulating monocytes and macrophages that infiltrate the perilesional area after ICH, and recombination efficiency in these peripheral populations is incomplete, an influx of cofilin-1-intact, non-recombined myeloid cells at the injury site could contribute disproportionately to the KO signal, particularly if KO lesions recruit more peripheral myeloid cells than Flx lesions. Third, we cannot exclude some degree of antibody cross-reactivity with the closely related isoforms cofilin-2 or ADF, which we did not control with isoform-specific blocking peptides in this experiment. We regard the second explanation, greater peripheral myeloid infiltration into larger KO lesions, as the most parsimonious given the larger hematoma volumes and elevated peripheral leukocyte changes we observe in KO mice (Fig. 8), but distinguishing definitively between these possibilities will require future studies using double immunofluorescence (e.g., IBA1/cofilin, NeuN/cofilin, GFAP/cofilin) or lineage-specific approaches will be valuable to identify the cellular origin of this response and to understand better how microglial cofilin-1 deficiency influences cofilin signaling in other cell types following ICH.

Peripheral blood plasma analyses revealed genotype-driven shifts in immune profiles, including altered counts of monocytes, lymphocytes, and neutrophil levels in the KO groups, suggesting that loss of microglial cofilin-1 disrupts the brain–immune interface. However, because constitutive CX3CR1-Cre mediates recombination in peripheral monocytes and macrophages in addition to microglia, the peripheral immune alterations observed in this study may reflect both direct effects of cofilin-1 deletion within peripheral myeloid cells and secondary immune responses to brain injury. Therefore, these findings should not be interpreted as being exclusively mediated by microglial dysfunction. These results contrast with our previous studies, which have shown that pharmacological inhibition or siRNA-mediated knockdown of cofilin-1 is neuroprotective after ICH [12, 13]. This discrepancy likely reflects transient inhibition after injury, driven by cofilin upregulation and accumulation of cofilin-actin rods, rather than chronic deletion at birth. Acute suppression of cofilin-1 may attenuate overactivation and oxidative stress by transiently blocking cofilin-actin rod formation and mitochondrial translocation [8]. However, lifelong deletion in microglia impairs essential homeostatic functions, including debris clearance and immune regulation. These findings highlight the importance of timing: whereas transient cofilin suppression mitigates post-ICH hyperactivation, chronic deficiency during birth primes microglia for over reactivity, possibly mediated by reduced TMEM119 expression, compensatory dysregulation of the cofilin signaling pathway (e.g., LIMK/SSH1), or an imbalance of the Rho GTPases. Our findings therefore further highlight that cofilin-1 exerts dual, context-dependent effects. While transient inhibition after acute ICH suppresses secondary injury cascades, sustained deficiency in microglia at baseline is detrimental and compromises microglial surveillance and repair functions. Future studies should investigate whether microglial dysfunction arises from disrupted actin turnover or secondary transcriptional reprogramming by exploring upstream regulators (LIMK/SSH1) or downstream effectors (cofilin rods after ICH). Additionally, sex differences observed in this study warrant investigation into hormonal regulation of microglial cofilin signaling, potentially through estrogen- or androgen receptor–mediated modulation of actin dynamics. Furthermore, Future studies warrant investigating phenotypic shifts using single-cell RNA-seq and conducting phagocytosis assays to elucidate the underlying mechanisms.

Finally, our findings have several potential translational implications. Previous studies have demonstrated beneficial effects of transient pharmacological inhibition of cofilin-1 during the acute phase of ICH [13]. Whereas our results indicate that chronic genetic deficiency of microglial cofilin-1 disrupts microglial homeostasis and worsens neurological outcomes. Together, these observations suggest that both the timing and extent of cofilin modulation are likely critical determinants of therapeutic efficacy. Although human studies have not yet established genetic associations between cofilin-1 and ICH outcomes, investigating genetic variation within the cofilin-1 signaling pathway, including upstream regulators such as LIMK and SSH phosphatases, may provide insight into interindividual susceptibility to secondary brain injury. Furthermore, the greater vulnerability observed in female knockout mice supports the growing recognition that sex-dependent immune mechanisms should be considered in the development of future therapeutic strategies targeting microglial cytoskeletal signaling.

## Limitation

Despite the strengths of our study, several limitations should be acknowledged. First, although the conditional cofilin-1 knockout was designed to target microglia, the constitutive CX3CR1-Cre driver used here is not restricted to microglia; it also recombines in peripheral monocytes, macrophages, and NK cells, and constitutive (non-inducible) CX3CR1-Cre lines have been reported to drive recombination in a subset of neurons [45, 46]. In addition, activation of CX3CR1-Cre-based systems has been reported to induce type I interferon signaling and associated microglial activation in the early postnatal brain [47]. We did not include Cre-only controls in the present cohort, nor did we directly quantify recombination efficiency in peripheral myeloid cells or assess cofilin-1 status in neurons or other non-microglial CNS populations; consequently, we cannot fully exclude a contribution of Cre-driven, cofilin-1-independent effects to the baseline phenotype, including the reduction in TMEM119 and the morphological changes we attribute to microglial cofilin-1 loss. Inducible, more restricted drivers such as Tmem119-CreER or P2ry12-CreER would allow this to be dissociated in future work [48]. Although all experimental groups were age-matched, the use of mature adult mice (6–8 months old) may influence baseline microglial activity and hormonal status compared with younger adult mice commonly used in experimental ICH studies.

We also employed only the collagenase-induced ICH model, which reliably mimics hematoma expansion but does not fully capture the heterogeneity of human ICH. Validation in alternative models, such as autologous blood injection or spontaneous hypertension-induced ICH, would provide broader insight and a more clinically relevant scenario. Moreover, our analyses were focused on the acute phase (day 3 post-ICH), leaving potential subacute and chronic consequences of microglial cofilin-1 deficiency unexplored. Behavioral findings, particularly the hyperlocomotion observed in female KO mice, should also be interpreted with caution, as additional assays are needed to disentangle disinhibition from altered anxiety or motivational states. Female mice were not monitored for estrous cycle at the time of behavioral testing. Because cycle stage is known to modulate locomotor activity, anxiety-like behavior, and neuroinflammatory responses, uncontrolled cycling could have contributed variance to, or masked more consistent effects within, the female behavioral phenotypes reported here, particularly the hyperlocomotion and reduced anxiety-like behavior observed in female KO mice. Future studies should incorporate vaginal cytology or another cycle-staging method to control this variable. AlthoughHet mice were included throughout our behavioral and post-ICH phenotyping and frequently showed intermediate outcomes, we did not directly quantify the degree of cofilin-1 protein or mRNA reduction in Het microglia relative to Flx and KO. The intermediate phenotypes we observe in Het mice are therefore consistent with, but do not directly confirm, a gene-dose relationship, and we recommend that future work include this quantification to formally establish the dose–response relationship implied by our behavioral and histological data.

Reduction in locomotor activity observed after ICH compared with baseline behavioral characterization is consistent with the neurological impairment induced by hemorrhagic brain injury. Because the same cohort of animals was assessed before and after ICH under identical testing conditions, the observed reduction primarily reflects the functional consequences of ICH. However, we did not include a sham surgery group (burr hole and needle tract without collagenase injection); therefore, we cannot fully exclude a contribution of surgical trauma itself to the microglial activation and behavioral changes observed after ICH, independent of hemorrhage. Future studies should incorporate a sham arm to isolate procedure-related from hemorrhage-specific neuroinflammation.

Mechanistically, while we demonstrate that cofilin-1 loss enhances microglial activation and worsens ICH outcomes, the upstream/downstream signaling pathways affecting actin remodeling, such as LIMK, SSH1, or Rho GTPase cascades, remain to be identified. Furthermore, although we observed apparent sex differences, sex hormone levels were not directly measured or manipulated, leaving open the question of whether the dimorphism reflects intrinsic microglial programming, hormonal modulation, or both. Finally, while our study was adequately powered for acute outcomes, larger cohorts, longer-term follow-up, and translational validation in human tissue will be required to strengthen the clinical relevance of these findings.

## Conclusion

In this study, we generated and characterized a novel microglia-specific cofilin-1 knockout model to explore how cytoskeletal regulation shapes microglial function under homeostatic and pathological conditions. Our results demonstrate that cofilin-1 deficiency disrupts microglial homeostasis, reduces TMEM119 expression, alters microglial morphology, and enhances microglial activation. Behavioral testing revealed sex-dependent alterations, with female knockout mice displaying heightened locomotor activity and exploratory behavior, and a decrease in anxiety-related behavior. Following ICH, microglial cofilin-1 deficiency increased hematoma volume, which was associated with cofilin-1 overexpression, microglial activation, and altered peripheral immune responses. Moreover, microglial cofilin-1 deficiency showed only a limited effect on brain water content or edema in the context of genotype. Importantly, these effects were more pronounced in females, suggesting a sex-specific vulnerability. Collectively, these findings establish cofilin-1 as a critical regulator of microglial homeostasis and neuroinflammatory responses, providing new insights into the cellular mechanisms underlying sex-related differences in outcomes of intracerebral hemorrhage.

## Supplementary Information

Below is the link to the electronic supplementary material.

Supplementary file1 (DOCX 2226 KB)

## Data Availability

No datasets were generated or analysed during the current study.
