# Supplementary material for: Microglial Cofilin-1 Regulates Neuroinflammatory Responses and Behavioral Outcomes Following Intracerebral Hemorrhage
Source: Mol Neurobiol. 2026 Nov 9;63(1):1024. doi: 10.1007/s12035-026-06324-7 (PMC13748338; doi:10.1007/s12035-026-06324-7)
Supplement: Supplementary file 1 [file 12035_2026_6324_MOESM1_ESM.docx]

Microglial Cofilin-1 Regulates Neuroinflammatory Responses and Behavioral Outcomes Following Intracerebral Hemorrhage

**Supplementary Figures and Tables**

**Supplementary Figure 1.** **Generation and genotyping of Cx3Cr1-specific cofilin-1 conditional knockout mice by crossbreeding CX3Cr1^Cre^ and cofilin^fl/fl^ (Flx).** **(A)** Schematic representation of the Cre/loxP breeding steps used to generate CX3Cr1^Cre^.cofilin^fl/wt^ (heterozygous; Het) mice and CX3Cr1^Cre^.cofilin^fl/fl^ (conditional knockout; KO) mice. The Cx3Cr1 promoter drives Cre recombinase expression, enabling deletion of the floxed cofilin-1 allele specifically in Cx3Cr1-expressing cells. **(B)** Representative PCR genotyping of ear punching DNA for Cofilin-1 (upper panel), showing wild-type (380 bp), floxed (470 bp), and deleted (170 bp) alleles, and for Cx3Cr1-Cre (lower panel), showing wild-type (302 bp) and Cre (380 bp) alleles. **(C)** Representative images of adult male and female Flx and KO mice show no obvious gross morphological differences between genotypes.

**Supplementary Figure 2.** **Body weight and plasma cofilin-1 concentration in male and female mice across three genotypes, including Flx (cofilin^fl/fl^), Het (CX3Cr1^Cre^.cofilin^fl/wt^), and KO (CX3Cr1^Cre^.cofilin^fl/fl^) mice.** (**A)** Body weight measurements for male and female mice across Flx, Het, and KO genotypes show no significant differences. Comparison between sexes reveals that male mice have significantly higher body weights than females within each genotype. **(B)** Plasma cofilin-1 concentrations in male and female mice show no significant genotype-dependent differences. A sex-based comparison of plasma cofilin-1 levels reveals higher concentrations in females for the Het and KO groups, but not in Flx mice. Data are presented as mean ± SEM, and statistical analysis was performed using two-way ANOVA followed by Tukey's multiple comparisons test (*p < 0.05, **p < 0.01, ****p < 0.0001), (n = 10- 14, animals per group per sex, as specified in each experiment).

**Supplementary Figure 3. Novel object recognition and spontaneous alternation performance in males and females in all genotypes in male and female floxed (Flx), heterozygous (Het), and knockout (KO) mice.** **(A)** Discrimination index in the novel object recognition test (NORT) showed no significant differences between genotypes in either sex. **(B)** The percentage of total investigation time in NORT was comparable across genotypes and sexes. **(C)** Percentage alternation in the T-maze spontaneous alternation task did not differ between genotypes or sexes. Data are presented as mean ± SEM; individual data points represent single animals. Statistical analysis was performed using two-way ANOVA followed by Tukey's multiple comparisons test (n = 10-14 animals per group per sex, as specified in each experiment).

**Supplementary Figure 4. Morphometric analysis of brain hemispheric and A–P length following cofilin-1 deletion in male and female floxed (Flx), heterozygous (Het), and knockout (KO) mice.** **(A–B)** Quantification of right and left hemispheric length in female **(A)** and male **(B)** mice across genotypes (Flx, Het, KO). Two-way ANOVA revealed significant effects of genotype in both sexes (female: *F*(2,24) = 3.586, *p* = 0.0434; male: *F*(2,24) = 5.257, *p* = 0.0128), with a significant right and left hemispheric length effect in both comparisons (female vs. male: *F*(1,24) = 163.0 and 72.73, respectively; *p* < 0.0001). Post hoc testing identified increased left hemispheric length in KO compared to Het males (*p* = 0.0300). **(C–D)** Direct comparison of right **(C)** and left **(D)** hemispheric lengths between male and female groups. Right hemisphere: significant main effects of genotype (*F*(2,24) = 3.910, *p* = 0.0339) and sex (*F*(1,24) = 6.925, *p* = 0.0146) without interaction; no post hoc pairwise differences reached significance. Left hemisphere: significant genotype effect (*F*(2,24) = 5.464, *p* = 0.0111) with KO males showing longer length than Het males (*p* = 0.0125). **(E)** A–P axis length was not significantly altered by genotype, sex, or their interaction (all *p* > 0.1). **(F–G)** Representative dorsal images of whole brains from male **(F)** and female **(G)** mice. **(H)** Schematic illustrating measurement strategy for right, left, and anterior–posterior (A–P) dimensions. Data are mean ± SEM; individual data points are shown for a single animal. *p* < 0.05, ***p* < 0.001, ****p* < 0.0001 by two-way ANOVA with Tukey's post hoc test (n = 5 animals per group per sex, as specified in each experiment).

**Supplementary Figure 5. Peripheral leukocyte profiles and plasma cofilin levels after intracerebral hemorrhage (ICH) in all genotypes (floxed (Flx), heterozygous (Het), and knockout (KO)) of male and female mice.** **(A)** Monocyte counts revealed a significant increase in female KO mice compared with Flx and Het. **(B)** Lymphocyte numbers were high in female KO mice relative to Flx. **(C)** Neutrophil counts were significantly lower in both male and female KO mice compared with Flx and Het controls. **(D)** Plasma cofilin concentrations, measured by ELISA, were unchanged across sex and genotype. Data are presented as mean ± SEM. Individual data points represent the results from a single mouse. Statistical analysis was performed using two-way ANOVA followed by Tukey's post hoc test; *P < 0.05, **P < 0.01 (n = 5-7 animals per group per sex, as specified in each experiment).

**Supplementary Table 1.** Primers used for PCR to confirm the genotype.

| **Gene** | **PCR** | **Primer ID** | **Sequence** |
| --- | --- | --- | --- |
| CFL1  (Cofilin-1) | fl | Primer1 | 5'- CGCTGGACCAGAGCACGCGGCATC -3' |
|  |  | Primer2 | 5'- CTGGAAGGGTTGTTACAACCCTGG -3' |
|  |  | Primer3 | 5'- CATGAAGGTTCGCAAGTCCTCAAC -3' |
| CX3cr1 | Cre | Common | 5'- GCA GGG AAA TCT GAT GCA AG -3' |
|  |  | Mutant | 5'- GAC ATT TGC CTT GCT GGA C -3' |
|  |  | WT | 5'- CCT CAG TGT GAC GGA GAC AG -3' |

**Supplementary Table 2.** Primers used for qPCR to characterize the KO mice.

| **Gene** | **Primer ID** | **Sequence** | **References** |
| --- | --- | --- | --- |
| 18S | Forward (Sense) | 5′-CCTGGATACCGCAGCTAGGA-3′ | [1] |
|  | Reverse (Antisense) | 5′-GCGGCGCAATACGAATGCCCC-3′ |  |
| CFL1  (Cofilin-1) | Forward (Sense) | 5′TCTGTCTCCCTTTCGTTTCC-3′ | [2] |
|  | Reverse (Antisense) | 5′CATTGAACACCTTGATGACACC-3′ |  |
| TMEM119 | KSPQ12012G | Predesigned SYBR Green Primer | Sigma Aldrich |

**Supplementary Table 3.** **Research Resource Identifiers (RRIDs) and Key Reagents**

| **Resource Type** | **Resource Name** | **Supplier** | **Identifier** |
| --- | --- | --- | --- |
| Mouse strain | Cofilin^fl/fl^ (floxed mice) | Provided by Marco B. Rust | RRID: N/A (custom strain) |
| Mouse strain | Cx3cr1^tm1.1(cre)Jung/J^ (Cre mice) | The Jackson Laboratory | RRID: IMSR_JAX:025524 |
| Software | Flow cytometry analysis software (FCS Express 7 Plus) | De Novo Software | RRID: SCR_016654 |
| Software | ImageJ | National Institutes of Health | RRID: SCR_003070 |
| Software | GraphPad Prism v10.5 | GraphPad Software | RRID: SCR_002798 |
| Software | AnyMaze behavioral tracking software | Stoelting Co. | RRID: SCR_014289 |
| Kit | Neural Dissociation Kit | Miltenyi Biotec | Cat. no. 130-092-628 |
| Reagent | Debris removal solution | Miltenyi Biotec | Cat. no. 130-109-398 |
| Reagent | CD11b (Microglia) MicroBeads | Miltenyi Biotec | Cat. no. 130-093-634 |
| Reagent | LS+ positive selection column | Miltenyi Biotec | Cat. no. 130-042-401 |
| Antibody | Anti-CD11b-PE | Miltenyi Biotec | Cat. no. 130-110-553 |
| Antibody | Anti-cofilin (primary antibody) | Abcam | Cat. no. ab54532 |
| Antibody | Goat anti-mouse IgG H&L (Alexa Fluor) | Abcam | Cat. no. ab150115 |
| Antibody | Anti-IBA1 (primary antibody) | FUJIFILM Wako Chemicals | Cat. no. 019-19741 |
| Secondary antibody | Alexa Fluor donkey anti-mouse IgG | Jackson ImmunoResearch | RRID: AB_2340851 |
| Secondary antibody | Texas Red Rabbit Anti-Horse IgG | Jackson ImmunoResearch | RRID: AB_2339610 |
| Reagent | 4′,6-Diamidino-2-Phenylindole (DAPI) | Fisher Scientific | Cat. no. NC9029229 |
| Reagent | TRIzol reagent | Fisher Scientific | Cat. no. 15596018 |
| Kit | iScript cDNA Synthesis Kit | Bio-Rad | Cat. no. 1708891 |
| Kit | SYBR Green Supermix | Bio-Rad | Cat. no. 1725271 |
| Kit | Mouse Cofilin ELISA Kit | Fisher Scientific | Cat. no. NBP275252 |
| Reagent | Paraformaldehyde (PFA) | Fisher Scientific | Cat. no. AC416780010 |
| Reagent | Phosphate-buffered saline (PBS) | Fisher Scientific | Cat. no. MT21031CV |
| Reagent | Bovine Serum Albumin | Fisher Scientific | Cat. no. 50-136-8067 |
| Equipment | BD FACSCanto Flow Cytometer (FACSCanto II System with Fluidics Cart (8-color, blue/red/violet) | BD Biosciences | Cat. no. 338962 |
| Equipment | VETSCAN HM5 Hematology Analyzer | Zoetis Inc. | Cat. no. 10023319 |
| Equipment | T100TM Thermal Cycler | Bio-Rad | Cat. no. 1861096 |
| Equipment | StepOne Plus Real-Time PCR System | Thermo Fisher Scientific | Cat. no. 4376598 |
| Equipment | Nikon Stereo Microscope | Mager Scientific | Cat. no. SMZ800N |
| Equipment | Nikon Fluorescence Microscope | Mager Scientific | Cat. no. TE2000-U |
| Equipment | Olympus VS120 Slide Scanner Slide Analyzer | Olympus Corporation | Cat. no. VS120 |

**Supplementary Table 4.** Two-way ANOVA results for motor coordination, strength, open field, novel object recognition test (NORT), and T-maze of floxed (Flx), heterozygous (Het), and knockout (KO) mice.

| **Tests** | **Two-way ANOVA** | | **Post hoc (Tukey's multiple comparisons test)** | | | |
| --- | --- | --- | --- | --- | --- | --- |
|  | **Male and Female** | **F (df), P value** | **Male** | **P value** | **Female** | **P value** |
| **Rotarod** | Interaction | F(2, 60) = 1.693, p = 0.1925 | Flx vs. Het | 0.8220 | Flx vs. Het | 0.2888 |
|  | Genotype | F(2, 60) = 0.2129, p = 0.8089 | Flx vs. KO | 0.9465 | Flx vs. KO | 0.9849 |
|  | Sex | F(1, 60) = 4.453, p = 0.0390 | Het vs. KO | 0.6173 | Het vs. KO | 0.3096 |
| **Grip Strength** | Interaction | F(2, 60) = 0.3255, p = 0.7234 | Flx vs. Het | 0.9652 | Flx vs. Het | 0.4446 |
|  | Genotype | F(2, 60) = 0.5529, p = 0.5782 | Flx vs. KO | 0.9491 | Flx vs. KO | 0.9441 |
|  | Sex | F(1, 60) = 12.82, p = 0.0007 | Het vs. KO | 0.9990 | Het vs. KO | 0.5813 |
| **Inverted Screen** | Interaction | F(2, 59) = 0.9517, p = 0.3919 | Flx vs. Het | 0.3777 | Flx vs. Het | 0.9797 |
|  | Genotype | F(2, 59) = 0.6297, p = 0.5363 | Flx vs. KO | 0.2136 | Flx vs. KO | 0.9874 |
|  | Sex | F(1, 59) = 2.749, p = 0.1026 | Het vs. KO | 0.9522 | Het vs. KO | 0.9982 |
| **Wire Hang** | Interaction | F(2, 60) = 0.6852, p = 0.5079 | Flx vs. Het | 0.9914 | Flx vs. Het | 0.4471 |
|  | Genotype | F(2, 60) = 18.73, p <0.0001 | Flx vs. KO | 0.0003 | Flx vs. KO | 0.0010 |
|  | Sex | F(1, 60) = 1.246, p = 0.2688 | Het vs. KO | 0.0005 | Het vs. KO | 0.0405 |
| **Open Field (Distance)** | Interaction | F(2, 60) = 1.290, p = 0.2829 | Flx vs. Het | >0.9999 | Flx vs. Het | 0.9926 |
|  | Genotype | F(2, 60) = 10.18, p = 0.0002 | Flx vs. KO | 0.2090 | Flx vs. KO | 0.0015 |
|  | Sex | F(1, 60) = 1.552, p = 0.2177 | Het vs. KO | 0.2076 | Het vs. KO | 0.0010 |
| **Open Field (Speed Average)** | Interaction | F(2, 60) = 1.731, p = 0.1859 | Flx vs. Het | 0.7256 | Flx vs. Het | 0.8041 |
|  | Genotype | F(2, 60) = 10.83, p < 0.0001 | Flx vs. KO | 0.1245 | Flx vs. KO | 0.0002 |
|  | Sex | F(1, 60) = 0.8210, p = 0.3685 | Het vs. KO | 0.4658 | Het vs. KO | 0.0021 |
| **Open Field (Time Mobile)** | Interaction | F(2, 59) = 2.348, p = 0.1044 | Flx vs. Het | 0.5376 | Flx vs. Het | 0.7351 |
|  | Genotype | F(2, 59) = 14.29, p < 0.0001 | Flx vs. KO | 0.0601 | Flx vs. KO | <0.0001 |
|  | Sex | F(1, 59) = 2.867, p = 0.0957 | Het vs. KO | 0.4393 | Het vs. KO | 0.0003 |
| **Open Field (Time in Center)** | Interaction | F(2, 60) = 2.581, p = 0.0841 | Flx vs. Het | 0.9972 | Flx vs. Het | 0.7107 |
|  | Genotype | F(2, 60) = 7.934, p = 0.0009 | Flx vs. KO | 0.5935 | Flx vs. KO | 0.0043 |
|  | Sex | F(1, 60) = 1.201, p = 0.2776 | Het vs. KO | 0.5473 | Het vs. KO | 0.0003 |
| **NORT (Discrimination Index)** | Interaction | F(2, 60) = 0.3360, p = 0.7160 | Flx vs. Het | 0.9368 | Flx vs. Het | 0.7577 |
|  | Genotype | F(2, 60) = 0.1278, p = 0.8802 | Flx vs. KO | 0.9590 | Flx vs. KO | 0.8980 |
|  | Sex | F(1, 60) = 0.5646, p = 0.4553 | Het vs. KO | 0.8014 | Het vs. KO | 0.9431 |
| **NORT (%Total Investigation Time)** | Interaction | F(2, 60) = 0.5071, p = 0.6048 | Flx vs. Het | 0.9089 | Flx vs. Het | 0.6371 |
|  | Genotype | F(2, 60) = 0.07452, p = 0.9283 | Flx vs. KO | 0.9946 | Flx vs. KO | 0.9282 |
|  | Sex | F(1, 60) = 0.2450, p = 0.6224 | Het vs. KO | 0.8550 | Het vs. KO | 0.8134 |
| **T-maze (%Alternation)** | Interaction | F(2, 61) = 0.3688, p = 0.6931 | Flx vs. Het | 0.8302 | Flx vs. Het | 0.1755 |
|  | Genotype | F(2, 61) = 1.485, p = 0.2345 | Flx vs. KO | 0.8927 | Flx vs. KO | 0.4031 |
|  | Sex | F(1, 61) = 2.663, p = 0.1079 | Het vs. KO | 0.9871 | Het vs. KO | 0.8046 |

**Supplementary Table 5.** Two-way ANOVA results for motor coordination, strength, neurological deficit score (NDS), and open field after intracerebral hemorrhage (ICH) across all genotypes, including floxed (Flx), heterozygous (Het), and knockout (KO) mice.

| **Tests** | **Two-way ANOVA** | | **Post hoc (Tukey's multiple comparisons test)** | | | |
| --- | --- | --- | --- | --- | --- | --- |
|  | **Male and Female** | **F (df), P value** | **Male** | **P value** | **Female** | **P value** |
| **Rotarod** | Interaction | F(2, 60) = 1.610, p = 0.2085 | Flx vs. Het | 0.0353 | Flx vs. Het | 0.0065 |
|  | Genotype | F(2, 60) = 14.58, p < 0.0001 | Flx vs. KO | 0.0001 | Flx vs. KO | 0.0169 |
|  | Sex | F(1, 60) = 1.473, p = 0.2297 | Het vs. KO | 0.1791 | Het vs. KO | 0.8203 |
| **Grip Strength** | Interaction | F(2, 59) = 2.236, p = 0.1159 | Flx vs. Het | 0.1170 | Flx vs. Het | 0.9910 |
|  | Genotype | F(2, 59) = 1.211, p = 0.3053 | Flx vs. KO | 0.9979 | Flx vs. KO | 0.6404 |
|  | Sex | F(1, 59) = 10.24, p = 0.0022 | Het vs. KO | 0.0865 | Het vs. KO | 0.7257 |
| **Inverted Screen** | Interaction | F(2, 60) = 1.291, p = 0.2825 | Flx vs. Het | 0.3417 | Flx vs. Het | 0.7965 |
|  | Genotype | F(2, 60) = 9.182, p = 0.0003 | Flx vs. KO | 0.0168 | Flx vs. KO | 0.0266 |
|  | Sex | F(1, 60) = 1.134, p = 0.2911 | Het vs. KO | 0.3660 | Het vs. KO | 0.0039 |
| **Wire Hang** | Interaction | F(2, 60) = 0.8592, p = 0.4287 | Flx vs. Het | 0.2427 | Flx vs. Het | >0.9999 |
|  | Genotype | F(2, 60) = 6.678, p = 0.0024 | Flx vs. KO | 0.0375 | Flx vs. KO | 0.0399 |
|  | Sex | F(1, 60) = 4.865, p = 0.0313 | Het vs. KO | 0.6894 | Het vs. KO | 0.0399 |
| **NDS** | Interaction | F(2, 60) = 0.1742, p = 0.8405 | Flx vs. Het | 0.7644 | Flx vs. Het | 0.9958 |
|  | Genotype | F(2, 60) = 1.047, p = 0.3573 | Flx vs. KO | 0.4036 | Flx vs. KO | 0.7788 |
|  | Sex | F(1, 60) = 0.002894, p = 0.9573 | Het vs. KO | 0.8399 | Het vs. KO | 0.7230 |
| **Open Field (Distance)** | Interaction | F(2, 59) = 2.744, p = 0.0726 | Flx vs. Het | 0.2089 | Flx vs. Het | 0.3202 |
|  | Genotype | F(2, 59) = 0.06150, p = 0.9404 | Flx vs. KO | 0.3070 | Flx vs. KO | 0.5749 |
|  | Sex | F(1, 59) = 8.194, p = 0.0058 | Het vs. KO | 0.9493 | Het vs. KO | 0.8527 |
| **Open Field (Speed Average)** | Interaction | F(2, 59) = 1.750, p = 0.1827 | Flx vs. Het | 0.2359 | Flx vs. Het | 0.6093 |
|  | Genotype | F(2, 59) = 0.2556, p = 0.7753 | Flx vs. KO | 0.3556 | Flx vs. KO | 0.9123 |
|  | Sex | F(1, 59) = 7.885, p = 0.0067 | Het vs. KO | 0.9412 | Het vs. KO | 0.8181 |
| **Open Field (Time Mobile)** | Interaction | F(2, 59) = 0.6859, p = 0.5076 | Flx vs. Het | 0.4139 | Flx vs. Het | 0.9447 |
|  | Genotype | F(2, 59) = 0.4149, p = 0.6623 | Flx vs. KO | 0.4494 | Flx vs. KO | >0.9999 |
|  | Sex | F(1, 59) = 20.92, p < 0.0001 | Het vs. KO | 0.9923 | Het vs. KO | 0.9332 |
| **Open Field (Time in Center)** | Interaction | F(2, 57) = 0.3967, p = 0.6744 | Flx vs. Het | 0.4122 | Flx vs. Het | 0.9999 |
|  | Genotype | F(2, 57) = 0.5231, p = 0.5955 | Flx vs. KO | 0.6677 | Flx vs. KO | 0.9090 |
|  | Sex | F(1, 57) = 18.58, p < 0.0001 | Het vs. KO | 0.8822 | Het vs. KO | 0.9150 |

**References**

1.	Kang, Y., et al., *Evaluation of reference genes for gene expression studies in mouse and N2a cell ischemic stroke models using quantitative real-time PCR.* BMC neuroscience, 2018. **19**(1): p. 3.

2.	Kanellos, G., et al., *ADF and Cofilin1 control actin stress fibers, nuclear integrity, and cell survival.* Cell reports, 2015. **13**(9): p. 1949-1964.
